# Quantitative comparison of microarray experiments with published leukemia related gene expression signatures

**DOI:** 10.1186/1471-2105-10-422

**Published:** 2009-12-15

**Authors:** Hans-Ulrich Klein, Christian Ruckert, Alexander Kohlmann, Lars Bullinger, Christian Thiede, Torsten Haferlach, Martin Dugas

**Affiliations:** 1 Department of Medical Informatics and Biomathematics, University of Münster, Domagkstraße 9, 48149 Münster, Germany; 2 Munich Leukemia Laboratory, Max-Lebsche-Platz 31, 81377 München, Germany; 3 Internal Medicine III, University of Ulm, Albert-Einstein-Allee 23, 89081 Ulm, Germany; 4 Medical Clinic I, University Hospital Dresden, Fetscherstraße 74, 01307 Dresden, Germany

## Abstract

**Background:**

Multiple gene expression signatures derived from microarray experiments have been published in the field of leukemia research. A comparison of these signatures with results from new experiments is useful for verification as well as for interpretation of the results obtained. Currently, the percentage of overlapping genes is frequently used to compare published gene signatures against a signature derived from a new experiment. However, it has been shown that the percentage of overlapping genes is of limited use for comparing two experiments due to the variability of gene signatures caused by different array platforms or assay-specific influencing parameters. Here, we present a robust approach for a systematic and quantitative comparison of published gene expression signatures with an exemplary query dataset.

**Results:**

A database storing 138 leukemia-related published gene signatures was designed. Each gene signature was manually annotated with terms according to a leukemia-specific taxonomy. Two analysis steps are implemented to compare a new microarray dataset with the results from previous experiments stored and curated in the database. First, the global test method is applied to assess gene signatures and to constitute a ranking among them. In a subsequent analysis step, the focus is shifted from single gene signatures to chromosomal aberrations or molecular mutations as modeled in the taxonomy. Potentially interesting disease characteristics are detected based on the ranking of gene signatures associated with these aberrations stored in the database. Two example analyses are presented. An implementation of the approach is freely available as web-based application.

**Conclusions:**

The presented approach helps researchers to systematically integrate the knowledge derived from numerous microarray experiments into the analysis of a new dataset. By means of example leukemia datasets we demonstrate that this approach detects related experiments as well as related molecular mutations and may help to interpret new microarray data.

## Background

Leukemia is a heterogeneous disease with respect to genetic alterations, which include chromosomal aberrations as well as molecular mutations. Thus far, microarray technology and in particular gene expression arrays have been widely used to explore the molecular variation underlying the biologic and clinical heterogeneity of leukemia [1]. As a result, biologically and clinically relevant subtypes of leukemia have been characterized based on their respective gene expression patterns [2-7]. Often, novel findings were published in the form of lists of differentially expressed genes that were referred to as gene expression signatures. When a new microarray dataset, herein denoted as query dataset, is analyzed, a thorough comparison with previously published results of similar experiments is helpful not only for verification, but also for identifying associations with different leukemia subtypes.

Solely relying on gene signatures, two microarray experiments can be compared by simply counting the number of overlapping genes from each study [8,9]. However, some studies reported limited overlap between lists of differentially expressed genes derived from different microarray studies for the same disease category [10-12]. Even when using technical replicates for inter- and intra-platform comparisons, the number of overlapping genes can be small [13]. The reason for these disappointing results is not necessarily originated in the quality of microarray technology itself, but rather in the percentage of overlapping genes as being considered as an unsuitable measurement for the reproducibility of microarray experiments [14]. Based on a statistical model, it has been shown that even in technical replicate tests using identical samples, it is highly possible that the lists of the most differentially expressed genes are very inconsistent [15,16]. Moreover, published gene signatures are derived from different laboratories, with study groups applying differing array platforms and using different statistical methods to generate gene lists of interest [17]. Hence, approaches [18,19] that compute the similarity of a given gene list with a collection of published gene signatures based on the number of overlapping genes are likely to miss relevant signatures.

If the microarray intensity values of the query dataset are available, the search for similar results in a gene signature database can be based directly on the continuous intensity data avoiding the need for counting overlapping genes. Many gene set analysis (GSA) methods for detecting differential expression in externally defined sets of genes have been proposed [20-22] and successfully applied to gain novel biological insights from microarray data [23,24]. The externally defined gene sets are usually derived from pathway databases or from the Gene Ontology [25] database, but rarely from published articles [23]. Although it has been shown that GSA methods can be useful for comparison of microarray experiments [26-28], only few databases contain published gene signatures [18,19,26,29]. These databases focus on published gene expression signatures of genetic and chemical perturbations and do not offer an exhaustive collection of results from of a certain research field like leukemia. Thus, to our knowledge, these methods were not yet used to systematically compare a new microarray dataset with previously published gene signatures.

Any query dataset and the published experiments can be compared directly based on their intensity values, assuming all required microarray raw datasets are available. For example, the connectivity map [30] is a database that consists of more than 500 gene expression profiles from human cell lines treated with perturbagens together with a pattern-matching algorithm that can be used to mine the database when a query dataset is given. A similar approach has recently been proposed to search the Gene Expression Omnibus array data repository [31] for related microarray experiments [32]. However, only a small number of experiments with a simple design on the same microarray platform were manually selected, reanalyzed and included into the search strategy. The difficulty of reanalyzing complex experiments [33,34] with limited annotation [35] and limited availability of raw data for historical microarray datasets, and considering intra- and interlaboratory as well as platform-dependent influences on data, impede the practical usage of such methods for an exhaustive search for similar experimental results.

In this paper, we present an approach for a quantitative comparison of a query dataset with published gene signatures. As a proof-of-concept design we focus on a database curated manually from numerous leukemia-related experiments using different microarray platforms. The approach is based on GSA methods together with an accurately annotated database including a simple taxonomy for leukemia subtypes. By means of investigating two exemplary datasets, we show that the approach is not only useful to verify published results, but also to detect putative linkages between different leukemia entities.

## Results and Discussion

### Outline

**Figure 1:**
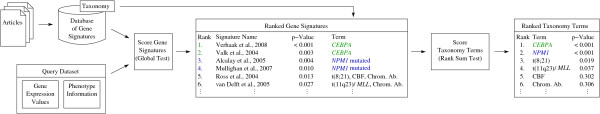
**Overview of the analysis process**. The proposed method relies on a manually curated database of leukemia-related published gene signatures annotated with terms from a predefined taxonomy. A new microarray dataset is analyzed in two steps. First, each signature is assessed by the global test method to constitute a ranking among the signatures. Secondly, the results from the first step are used to assess terms from the leukemia taxonomy that represent leukemia-related genetic aberrations and molecular mutations.

Figure 1 provides an overview of the proposed quantitative literature review process. First, the query dataset including the normalized microarray data as well as the studied phenotype variable has to be provided. For each gene signature in our database, the expression values of the signature's genes are read in the query dataset and scored, using the global test method [36], by their ability to explain the phenotypic variable of the query dataset. The resulting ranking of signatures, together with the association between taxonomy terms and signatures, is finally used to assess terms from our manually defined leukemia taxonomy. We implemented our approach as a web application [37] with special attention to visualization and exploratory representation of the results.

We initially had considered data from The Minimum Information about a Microarray Experiment (MIAME) standard [38], together with the MGED Ontology [39] to focus on an adequate description on how microarray data was obtained including many details about laboratory protocols, array design and samples used. However, these standards do not necessarily define a common format for a user-friendly description of gene signatures [40] and were therefore of limited usefulness for the construction of our database.

### Database for published leukemia gene signatures

A manually curated and annotated database storing published gene signatures in a computer processable format is essential for the presented approach. Currently, our database contains 138 leukemia-related gene signatures that were manually selected from 37 published research articles [Additional file 1: Supplemental Table S1]. These 138 signatures contain overall approximately 18000 entries of accession numbers and microarray probe set identifiers, representing nearly 6000 different genes (Table 1).

**Table 1:** Content of the data base for leukemia gene signatures.

	Number of signatures	Number of entries
Diagnostic	112	16748

Prognostic	8	646

Other	18	867

Overall	138	18261

138 gene signatures are stored in the database. The size of each signature varies between 10 and a few hundred accession numbers.

All gene signatures are stored as a collection of platform-independent accession numbers from the GenBank database [41], thus representing the detected mRNA-sequences in a given experiment. This process outperforms gene-centric approaches based on gene identifiers like HGNC's gene symbols, since transcripts that have not been assigned to any approved gene symbol yet can be stored. In addition, the assignment of transcripts to genes may undergo changes over time that can be better tracked when accession numbers are used. Few gene signatures [42,43] neither specifying accession numbers nor manufacturer specific microarray design identifiers that can be mapped to accession numbers are omitted. Accession numbers are regularly mapped to gene symbols using Entrez Gene and UniGene databases [44].

For interpretation of a gene signature it is necessary to store information about the underlying microarray experiment. Depending on the objective of the respective microarray study assessed for inclusion into the database, we distinguished between three types of signatures in our leukemia concept database. *Diagnostic *signatures report genes that are differentially expressed between two or more leukemia subtypes and thus can be used to discriminate certain disease categories. For instance, a gene signature used to discriminate between acute lymphoblastic leukemia (ALL) samples with different karyotypes [3] falls into this category. *Prognostic *signatures contain genes that are correlated with reported survival times [45]. The last type is a remainder group called *Other*, e.g., including a signature from a study reporting gene expression changes associated with certain treatment regimens [46,47]. In addition to this basic classification, we described the characteristics of the patient samples that were studied in the microarray experiment by means of a specific taxonomy for leukemia subtypes. The taxonomy was constructed by successively adding leukemia entities and mutations that were studied by experiments in our database. It consists of three hierarchies. The first one describes different major groups of leukemia based on the affected cell lineages (lymphoid, myeloid). The second one describes chromosomal aberrations (e.g. translocations, inversions), while the third one describes single gene mutations (e.g. *NPM1*, *CEBPA*).

### Assessment of gene signatures

A gene signature is considered potentially interesting, if the expression values of its genes in the query dataset are associated with the phenotype variable. Many different methods have been proposed to detect such sets of genes [20-22]. For our application, the global test method [48] was selected to test the self-contained null hypothesis [49] for each signature in our database. The resulting *p*-values are adjusted by Holm's method [50] to control the Family Wise Error Rate (FWER) and are used to rank the signatures. Informally, the self-contained null hypothesis states that there is no correlation in the query dataset between the phenotype variable and the gene expression values of any gene in the given signature. Hence, the self-contained null hypothesis is very restrictive. In terms of reproducibility of a gene signature, its rejection can be considered as a minimal requirement. However, the resulting *p*-values are a sensible criterion for ranking gene signatures.

The global test has been specified for categorical as well as continuous phenotype variables including right censored survival times [36,51] so that gene signatures can be assessed in a similar way independent of the scale of measurement of the phenotype variable. In contrast to many other GSA methods, a parametric approximation of the null distribution of the global test's test statistic is available. Hence, the computational effort of testing all signatures is small and we thus implemented our approach as a web application.

**Figure 2:**
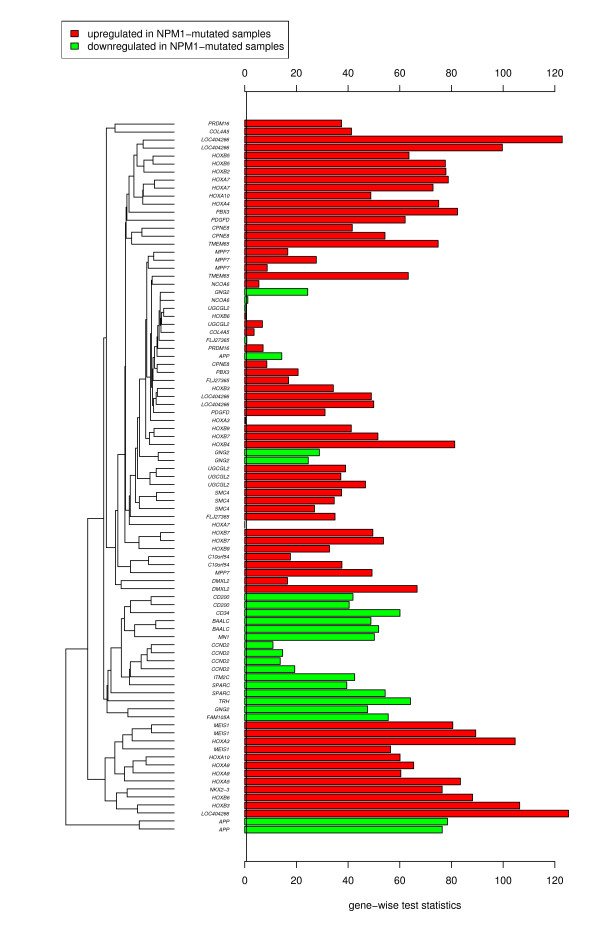
**NPM1 gene signature from Verhaak et al**. Verhaak et al. [57] published a *NPM1 *signature of 68 accession numbers that correspond to 40 genes. These genes were measured by 89 probe sets in the query dataset. A bar is plotted for each probe representing the value contributed by that probe set to the global test statistic. The expectation of these values under the null hypothesis of no correlation between *NPM1 *status and gene expression in the query dataset is indicated by the vertical black line. Overall, most genes reported by Verhaak et al. were also highly correlated with the *NPM1 *mutation status in our dataset. The colors indicate the direction of regulation. E.g., *CD200 *and *BAALC *were downregulated in *NPM1*-mutated samples, while most of the *HOXA@ *and *HOXB@ *genes showed increased expression in *NPM1*-mutated AML samples with a normal karyotype.

**Figure 3:**
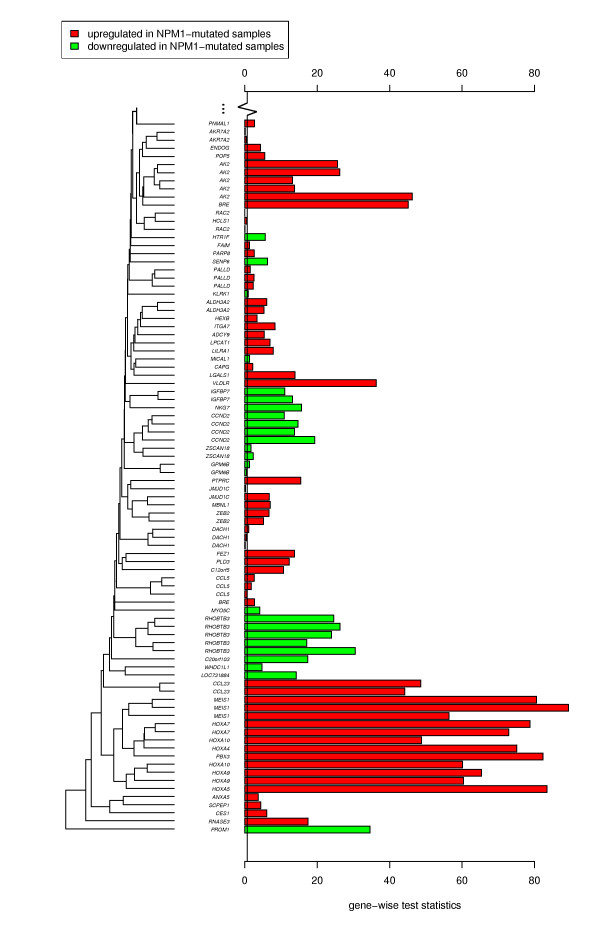
**t(11q23)/MLL gene signature from Ross et al**. Gene-wise test statistics are shown for a subset of 85 probe sets allocated to genes as reported by Ross et al. [59] to be associated with translocation t(11q23)/*MLL*. The full plot with all 185 probe sets that could be mapped to the signature from Ross et al. (100 accession numbers) is provided online [Additional file 1: Supplemental Figure S1]. The high correlation of the expression pattern of the Ross et al. signature with the *NPM1 *status in the query dataset was mainly caused by the *TALE *genes (*MEIS1 *and *PBX3*) and by some *HOXA@ *family genes. This was characteristic for the t(11q23)/*MLL *signatures in our database and is consistent with results reported in [61].

The *p*-values derived from the global test are related to signatures as a whole. For subsequent interpretation it is often useful to inspect which genes within a signature contributed mostly to the test result. Goeman et al. [36] used a decomposition of the global test statistic into gene-wise statistics as shown in equation (1) to generate informative plots. We adapted these plots and further ordered the genes by hierarchical clustering with Euclidean distance and average linkage so that groups of genes that strongly influenced the test statistic can be easily identified (shown in Figures 2 and 3). The gene-wise statistics are divided by their standard deviation and plotted as horizontal bars. The black vertical line indicates their expectation under the null hypothesis.

### Assessment of taxonomy terms

After assessment of all gene signatures in the database, the focus is shifted from single experiments to leukemia subtypes and their underlying genetic mutations that are modeled in the leukemia taxonomy. A taxonomy term can be considered of relevance with respect to the query dataset, if the ranks of the gene signatures associated with that term are low. For each term, the one-sided Mann-Whitney U-test is used to compare the ranks of associated signatures to the ranks of all other signatures. Due to the explorative nature of these tests, the resulting *p*-values are not adjusted for multiple testing. Depending on whether the differentiation of leukemia subtypes or survival times were studied in the query dataset, only *Diagnostic *signatures or *Prognostic *signatures were used for the described assessment of taxonomy terms.

The taxonomy terms together with the *p*-values are useful for exploring relations between the studied phenotype in the query dataset and leukemia subtypes with their specific mutations based on the knowledge gathered from many microarray studies. A low *p*-value of a term indicates that genes known to be associated with the leukemia entity represented by the term are correlated with the phenotype variable in the given query dataset.

### Example analyses

#### Differential expression between acute leukemia types

Golub et al. [2] published a signature of 50 genes that were differentially expressed between acute myeloid leukemia (AML) and acute lymphoblastic leukemia (ALL). Following that approach, van Delft et al. [52] studied differences in gene expression between pediatric AML and ALL patient samples and also presented a list of differentially expressed genes. About their gene signature, van Deft et al. stated: *"This gene list is almost entirely different from a previously published set of genes that discriminate ALL from AML (Golub et al.), with only LYN and ARHG in common between these two lists." *However, they demonstrated that their AML and ALL samples could be distinguished based on the Golub et al. signature, thus any missing agreement of their results was probably due to the number of overlapping genes being an unsuitable measurement.

To run our literature comparison, the dataset from van Delft et al. (59 ALL and 18 AML samples) was normalized using the Variance-Stabilizing Normalization method [53] and used as query dataset in the following example. Besides the gene signature described above, in their accompanying supplemental material van Delft et al. published an additional gene signature discriminating AML from ALL generated by using a different statistical gene selection method. These two signatures as well as the Golub et al. signature are the sole AML/ALL gene signatures stored in our database. Applying the global test to all gene signatures based on the dataset from van Delft et al. with the AML/ALL status as phenotype variable leads to many small *p*-values, because differences in gene expression between AML and ALL are distinctive. Nevertheless, the ranking remains reasonable. The signatures from van Delft et al. occupied the ranks one and three while the signature obtained by Golub and colleagues had the second position [Additional file 1: Supplemental Table S2]. The taxonomy terms "ALL" and "AML" had the first and second rank [Additional file 1: Supplemental Table S3].

Although van Delft et al. used a different microarray platform than Golub et al. and the overlap of the gene signatures was small, our GSA based approach successfully detected the signature from Golub et al. as potentially interesting and placed it at the second position. Intriguingly, one signature published in the article by van Delft et al. [52] and thus generated from the query dataset itself ranked below the Golub et al. signature. That may be caused by the impact of different data analysis procedures on gene lists [17,54]. Differences between adult and pediatric patients seem not to have a strong influence in this analysis.

#### NPM1 mutation in AML with normal karyotype

In a recent multi-center study, 251 gene expression profiles of AML specimens with normal karyotype were generated to delineate differential gene expression signatures corresponding to distinct gene mutations [55]. 138 of the 251 cases had a confirmed nucleophosmin gene (*NPM1*) mutation. The raw data can be accessed through the Gene Expression Omnibus database [31] (GSE15434). We applied the Robust Multichip Average algorithm [56] to normalize the data. Then, we used our approach to compare the differences in gene expression between *NPM1*-mutated and *NPM1 *wild type cases observed in this new dataset with previously reported results from our database.

First, all 138 gene signatures in the database were assessed by the global test and ranked according to their *p*-values. 8 signatures were annotated with the term "*NPM1 *mutated" from our taxonomy. These 8 signatures ranked among the first 21 signatures as shown in Table 2 and had highly significant *p*-values (FWER < 0.01) due to the large number of samples and the strength of changes in gene expression induced by *NPM1 *mutations. The first ranked signature was a *NPM1 *signature published by Verhaak et al. [57]. Figure 2 shows the contribution of single genes reported by Verhaak et al. on the global test result. In particular, many reported *HOXA *and *HOXB *family cluster genes as well as *MEIS1 *were also highly correlated with the *NPM1 *status in our query dataset.

**Table 2:** Ranking of gene signatures.

Rank	Gene signature	Taxonomy terms
1	Verhaak et al., Haematologica, 2009, AML, *NPM1*	*NPM1 *mutated

2	Verhaak et al., Haematologica, 2009, AML, *NPM1 *and *FLT3*-ITD	-

3	Verhaak et al., Haematologica, 2009, AML, *NPM1 *without *FLT3*-ITD	*NPM1 *mutated

4	Verhaak et al., Haematologica, 2009, AML, *FLT3*-ITD or *FLT3*-TKD	*FLT3*

5	Alcalay et al., Blood, 2005, AML, *NPM1*	*NPM1 *mutated

6	Verhaak et al., Haematologica, 2009, AML, *FLT3*-ITD	*FLT3*-ITD, *FLT3*

7	Alcalay et al., Blood, 2005, AML, *NPM1*,	*NPM1 *mutated

8	Valk et al., N Engl J Med, 2004, Classification of AML subtypes	-

9	Ross et al., Blood, 2004, AML and ALL, t(11q23)/*MLL*	t(11q23)/*MLL*, Chrom. aberration

10	Mullighan et al., Leukemia, 2007, AML, *NPM1*	*NPM1 *mutated

11	Mullighan et al., Leukemia, 2007, AML, *NPM1*	*NPM1 *mutated

12	Verhaak et al., Haematologica, 2009, AML, del(7q)	del(7q)

13	Mullighan et al., Leukemia, 2007, AML, *NPM1*	*NPM1 *mutated

14	Verhaak et al., Haematologica, 2009, AML, t(15;17)	t(15;17), Chrom. aberration

15	Marcucci et al., J Clin Oncol, 2008, AML, *CEBPA*	*CEBPA*

16	Stirewalt et al., Genes Chromosomes Cancer, 2008, AML	AML, Leukemia

17	Valk et al., N Engl J Med, 2004, AML, *CEBPA*	*CEBPA*

18	Ross et al., Blood, 2003, B-ALL, t(11q23)/*MLL*	-

19	van Delft et al., Br J Haematol, 2005, AML, t(11q23)/*MLL*	t(11q23)/*MLL*, Chrom. aberration

20	Valk et al., N Engl J Med, 2004, AML, cluster without predominant characteristics	-

21	Verhaak et al., Blood, 2005, AML, *NPM1*	*NPM1 *mutated

22	Langer et al., Blood, 2008, AML, *BAALC*	-

23	van Delft et al., Br J Haematol, 2005, AML, t(11q23)/*MLL*	t(11q23)/*MLL*, Chrom. aberration

24	Armstrong et al., Nat Genet, 2002, ALL, t(11q23)/*MLL*	t(11q23)/*MLL*, Chrom. aberration

25	Valk et al., N Engl J Med, 2004, AML, mostly *EVI1*	-

⋮	⋮	⋮

Each of the 138 gene signatures was tested for differential expression between *NPM1*-mutated and *NPM1 *wild type cases in the query dataset and ranked according to its *p*-value. All 8 of the 138 signatures associated with the taxonomy term "*NPM1 *mutated" ranked among the first 21 positions. The complete ranking of all signatures is available in the supplement [Additional file 1: Supplemental Table S4].

The result of the subsequent assessment of taxonomy terms is given in Table 3. Besides the *NPM1 *mutation, the translocation t(11q23)/*MLL *on the second-ranked position also had a noticeable low *p*-value [58]. This indicates that t(11q23)/*MLL *partially affects the differential expression of the same genes as the *NPM1 *mutation in our query dataset. Figure 3 provides an insight into the expression patterns of the genes reported by Ross et al. [59] (rank 9). Apparently, the differential expression of some *HOX *family cluster genes were affected by both the *MLL *gene rearrangement and the *NPM1 *mutations. Thus, we verified the published *NPM1 *signatures by means of the new dataset and detected potentially new associations to other chromosomal aberrations as well.

**Table 3:** Ranking of taxonomy terms.

Rank	unadjusted *p*-value	Term	Number of signatures	Number of articles
1	< 0.001	*NPM1 *mutated	8	4

2	0.028	t(11q23)/*MLL*	9	6

3	0.071	*CEBPA*	7	5

4	0.087	del(7q)	1	1

5	0.113	*FLT3*	6	3

⋮	⋮	⋮	⋮	⋮

Taxonomy terms were assessed based on the ranking of the gene signatures associated with those terms. In case of the example dataset examining *NPM1*-mutations, the 8 *NPM1 *signatures that were extracted from 4 different articles significantly occupied low ranks. The low *p*-value of translocation t(11q23)/*MLL *indicates a putative relation between this translocation and the studied *NPM1*-mutation. The full ranking of all taxonomy terms is provided in the supplement [Additional file 1: Supplemental Table S5]. The ranking remained reasonably stable when (i) half of the arrays were excluded from the analysis [Additional file 1: Supplemental Figure S2] and also when (ii) half of the gene signatures were excluded from the analysis [Additional file 1: Supplemental Figure S3].

## Conclusions

The presented approach allows a comprehensive and quantitative comparison of experimental microarray data with previously published results across different array platforms and microarray designs. The database is designed as an open structure to be independent from microarray manufacturer or differing chip designs. By means of an exemplary research topic, i.e. differential gene expression in leukemia subtypes, we have demonstrated that the approach is not only useful to verify published results but may also detect novel associations between genetic aberrations and gene mutations that affect the same biological processes and cellular pathways. It is expected that the same approach can also be extended to other areas of interest, such as querying signatures for other cancer types, microRNAs, DNA or histone methylation of promoter regions, or distinct signaling pathways. An implementation of our approach as well as the database itself and further example analyses are freely available on our website [37].

## Methods

### Global test

Goeman et al. give a general derivation of their global test as a score test in [48]. Here, the phenotype variable was binary in both presented examples, so that the global test could be specified within a logistic regression model as in [36] with test statistic

*X *= (*x*_*ij*_) denotes a *n *× *m *matrix of gene expression values of *n *arrays and *m *genes (all genes from the query dataset that are elements of the tested gene signature). *y *is the vector of the *n *observed phenotypes. *μ *is the expectation and *σ *the standard deviation of the phenotype variable, which are supposed to be known in this section. *S *has expectation *ES *= *tr*(*XX*^*t*^) and variance *VarS *≈ *tr*(*XX*^*t*^)^2 ^under the null hypothesis. *S *can be written as a sum of gene-wise terms:

For better comparability these *m *gene-wise terms were divided by their standard deviation before they were used to create the bar plots shown in Figures 2 and 3.

The null distribution of *S *is approximated by a series expansion in chi-square distribution functions as implemented in the R package globaltest [36]. Very small *p*-values (< 10^-12^) may not be numerically reliable. For that reason, only *p*-values up to 10^-12 ^are used to rank signatures whereas the standardized test statistic  = (*S *- *ES*)/*VarS *is used to rank remaining gene signatures with *p *< 10^-12^.

### Mann-Whitney U-test

The assessment of the taxony terms is based on the ranking derived from the global test of all *n *= 112 *Diagnostic *signatures. Let *r*_*i *_denote the rank of the *i*-th signature and *T*_*j *_is the set of all signatures associated with taxonomy term *t*_*j*_. For each term in our taxonomy with at least one associated gene signature, the one-sided Mann-Whitney U-test is used to test the null hypothesis, that the distribution of the global test's *p*-values of the signatures in *T*_*j *_differ by a location shift of *c *≥ 0 from the distribution of *p*-values of the signatures that are not in *T*_*j*_. The test statistic *W*_*j *_=  is standardized and a normal approximation with continuity correction is used to calculate *p*-values. This results in 34 dependet *p*-values, which are not corrected for multiple testing due to the explorative nature of the taxonomy analysis.

### Implementation

We used Axis2 to implement a Web Service interface to our PostgreSQL database that stores the gene signatures. The taxonomy was modeled in the Web Ontology Language (OWL). All tests were computed within R/Bioconductor [60] and the globaltest package [36]. Java Server Pages were used to realize the web-based graphical user interface.

## Authors' contributions

HUK designed and implemented the method, wrote the manuscript and analyzed results. CR participated in the design of the method and implemented the method. AK analyzed results, improved the method, and contributed to writing the manuscript. LB, CT and TH performed research and interpreted results. MD improved the design and analyzed results. All authors read and approved the final manuscript.

## Supplementary Material

Additional file 1 **Supplementary information**. Detailed information about the content of the leukemia gene signature database, complete rankings of gene signatures and taxonomy terms for both example analyses presented in the article and additional information about the stability of the taxonomy term ranking. Click here for file
