# Supplementary material for: Quantitative comparison of microarray experiments with published leukemia related gene expression signatures
Source: BMC Bioinformatics. 2009 Dec 15;10:422. doi: 10.1186/1471-2105-10-422 (PMC2803858; doi:10.1186/1471-2105-10-422)
Supplement: Additional file 1 — Supplementary information. Detailed information about the content of the leukemia gene signature database, complete rankings of gene signatures and taxonomy terms for both example analyses presented in the article and additional information about the stability of the taxonomy term ranking. [file 1471-2105-10-422-S1.PDF]

Quantitative comparison of microarray experiments with published leukemia related gene expression signatures

Supplementary information

Table S1 - Articles selected for the gene signature database	2
Table S2 - Differential expression between AML and ALL: Ranking of gene signatures	6
Table S3 - Differential expression between AML and ALL: Ranking of taxonomy terms	15
Table S4 - *NPM1* mutation in AML with normal karyotype: Ranking of gene signatures	16
Table S5 - *NPM1* mutation in AML with normal karyotype: Ranking of taxonomy terms	25
Figure S1 - t(11q23)/*MLL* gene signature from Ross et al.	26
Figure S2 - Stability of taxonomy term ranking – Sampling arrays	27
Figure S3 - Stability of taxonomy term ranking – Sampling gene signatures	28

Table S1 - Articles selected for the gene signature database

138 gene expression signatures were extracted from 37 manually selected articles and stored in the database for leukemia related signatures. This table lists all articles together with the number of signatures derived from each article.

Article	Number of signatures
Vey N, Mozziconacci MJ, Bertucci F et al. **Identification of new classes among acute myelogenous leukaemias with normal karyotype using gene expression profiling.** *Oncogene 2004*, 23(58):9381-91	5
Yeoh EJ, Ross ME, Downing JR et al. **Classification, subtype discovery, and prediction of outcome in pediatric acute lymphoblastic leukemia by gene expression profiling.** *Cancer Cell 2002*, 1(2):133-43	7
Wouters BJ, Lowenberg B, Delwel R et al. **Double *CEBPA* mutations, but not single *CEBPA* mutations, define a subgroup of acute myeloid leukemia with a distinctive gene expression profile that is uniquely associated with a favorable outcome.** *Blood 2009*, 113(13):3088-91	2
Verhaak RG, Goudswaard CS, Valk PJ et al. **Mutations in nucleophosmin (*NPM1*) in acute myeloid leukemia (AML): association with other gene abnormalities and previously established gene expression signatures and their favorable prognostic significance.** *Blood 2005*, 106(12):3747-54	1
Whitman SP, Ruppert AS, Bloomfield CD et al. ***FLT3* D835/I836 mutations are associated with poor disease-free survival and a distinct gene-expression signature among younger adults with de novo cytogenetically normal acute myeloid leukemia lacking *FLT3* internal tandem duplications.** *Blood 2008*, 111(3):1552-9	1
Hofmann WK, de Vos S, Ottmann OG et al. **Relation between resistance of Philadelphia-chromosome-positive acute lymphoblastic leukaemia to the tyrosine kinase inhibitor STI571 and gene-expression profiles: a gene-expression study.** *Lancet 2002*, 359(9305):481-6	2
Verhaak RG, Wouters BJ, Valk PJ et al. **Prediction of molecular subtypes in acute myeloid leukemia based on gene expression profiling.** *Haematologica 2009*, 94(1):131-4	17
Ross ME, Mahfouz R, Downing JR et al. **Gene expression profiling of pediatric acute myelogenous leukemia.** *Blood 2004*, 104(12):3679-87	8
Kohlmann A, Schoch C, Haferlach T et al. **Pediatric acute lymphoblastic leukemia (ALL) gene expression signatures classify an independent cohort of adult ALL patients.** *Leukemia 2004*, 18(1):63-71	1

Ebert BL, Galili N, Raza A et al.<br>**An erythroid differentiation signature predicts response to lenalidomide in myelodysplastic syndrome.**<br>*PLoS Med 2008*, 5(2):e35	1
Valk PJ, Verhaak RG, Delwel R et al.<br>**Prognostically useful gene-expression profiles in acute myeloid leukemia.**<br>*N Engl J Med 2004*, 350(16):1617-28	16
Cheok MH, Yang W, Evans WE et al.<br>**Treatment-specific changes in gene expression discriminate in vivo drug response in human leukemia cells.**<br>*Nat Genet 2003*, 34(1):85-90	10
Virtaneva K, Wright FA, Krahe R et al.<br>**Expression profiling reveals fundamental biological differences in acute myeloid leukemia with isolated trisomy 8 and normal cytogenetics.**<br>*Proc Natl Acad Sci U S A 2001*, 98(3):1124-9	4
Yagi T, Morimoto A, Ichikawa H et al.<br>**Identification of a gene expression signature associated with pediatric AML prognosis.**<br>*Blood 2003*, 102(5):1849-56	1
Bullinger L, Döhner K, Pollack JR et al.<br>**Use of gene-expression profiling to identify prognostic subclasses in adult acute myeloid leukemia.**<br>*N Engl J Med 2004*, 350(16):1605-16	1
van Delft FW, Bellotti T, Saha V et al.<br>**Prospective gene expression analysis accurately subtypes acute leukaemia in children and establishes a commonality between hyperdiploidy and t(12;21) in acute lymphoblastic leukaemia.**<br>*Br J Haematol 2005*, 130(1):26-35	18
Golub TR, Slonim DK, Lander ES et al.<br>**Molecular classification of cancer: class discovery and class prediction by gene expression monitoring.**<br>*Science 1999*, 286(5439):531-7	1
Schoch C, Kohlmann A, Haferlach T et al.<br>**Acute myeloid leukemias with reciprocal rearrangements can be distinguished by specific gene expression profiles.**<br>*Proc Natl Acad Sci U S A 2002*, 99(15):10008-13	2
Mullighan CG, Kennedy A, Downing JR et al.<br>**Pediatric acute myeloid leukemia with *NPM1* mutations is characterized by a gene expression profile with dysregulated *HOX* gene expression distinct from *MLL*-rearranged leukemias.**<br>*Leukemia 2007*, 21(9):2000-9	5
Marcucci G, Baldus CD, Bloomfield CD et al.<br>**Overexpression of the ETS-related gene, *ERG*, predicts a worse outcome in acute myeloid leukemia with normal karyotype: a Cancer and Leukemia Group B study.**<br>*J Clin Oncol 2005*, 23(36):9234-42	1

Kohlmann A, Schoch C, Haferlach T et al.<br>**Molecular characterization of acute leukemias by use of microarray technology**.<br>*Genes Chromosomes Cancer 2003*, 37(4):396-405	2
Debernardi S, Lillington DM, Young BD et al.<br>**Genome-wide analysis of acute myeloid leukemia with normal karyotype reveals a unique pattern of homeobox gene expression distinct from those with translocation-mediated fusion events.**<br>*Genes Chromosomes Cancer 2003*, 37(2):149-58	1
Lindvall C, Furge K, Teh BT et al.<br>**Combined genetic and transcriptional profiling of acute myeloid leukemia with normal and complex karyotypes.**<br>*Haematologica 2004*, 89(9):1072-81	1
Alcalay M, Tiacci E, Pelicci PG et al.<br>**Acute myeloid leukemia bearing cytoplasmic nucleophosmin (NPMc+ AML) shows a distinct gene expression profile characterized by up-regulation of genes involved in stem-cell maintenance.**<br>*Blood 2005*, 106(3):899-902	2
Metzeler KH, Hummel M, Buske C et al.<br>**An 86-probe-set gene-expression signature predicts survival in cytogenetically normal acute myeloid leukemia.**<br>*Blood 2008*, 112(10):4193-201	1
Wouters BJ, Jordà MA, Delwel R et al.<br>**Distinct gene expression profiles of acute myeloid/T-lymphoid leukemia with silenced *CEBPA* and mutations in *NOTCH1*.**<br>*Blood 2007*, 110(10):3706-14	1
Marcucci G, Maharry K, Bloomfield CD et al.<br>**Prognostic significance of, and gene and microRNA expression signatures associated with, *CEBPA* mutations in cytogenetically normal acute myeloid leukemia with high-risk molecular features: a Cancer and Leukemia Group B Study.**<br>*J Clin Oncol 2008*, 26(31):5078-87	1
Stirewalt DL, Meshinchi S, Radich JP et al.<br>**Identification of genes with abnormal expression changes in acute myeloid leukemia.**<br>*Genes Chromosomes Cancer 2008*, 47(1):8-20	2
Langer C, Radmacher MD, Bloomfield CD et al.<br>**High *BAALC* expression associates with other molecular prognostic markers, poor outcome, and a distinct gene-expression signature in cytogenetically normal patients younger than 60 years with acute myeloid leukemia: a Cancer and Leukemia Group B (CALGB) study.**<br>*Blood 2008*, 111(11):5371-9	1
Ferrando AA, Neuberg DS, Look AT et al.<br>**Gene expression signatures define novel oncogenic pathways in T cell acute lymphoblastic leukemia.**<br>*Cancer Cell 2002*, 1(1):75-87	3

Bhojwani D, Kang H, Carroll WL et al.<br>**Gene expression signatures predictive of early response and outcome in high-risk childhood acute lymphoblastic leukemia: A Children's Oncology Group Study.**<br>*J Clin Oncol 2008*, 26(27):4376-84	2
Lacayo NJ, Meshinchi S, Dahl GV et al.<br>**Gene expression profiles at diagnosis in de novo childhood AML patients identify *FLT3* mutations with good clinical outcomes.**<br>*Blood 2004*, 104(9):2646-54	3
Ross ME, Zhou X, Downing JR et al.<br>**Classification of pediatric acute lymphoblastic leukemia by gene expression profiling.**<br>*Blood 2003*, 102(8):2951-9	7
Pellagatti A, Cazzola M, Boultwood J et al.<br>**Gene expression profiles of CD34+ cells in myelodysplastic syndromes: involvement of interferon-stimulated genes and correlation to FAB subtype and karyotype.**<br>*Blood 2006*, 108(1):337-45	2
Boultwood J, Pellagatti A, Wainscoat JS et al.<br>**Gene expression profiling of CD34+ cells in patients with the 5q-syndrome.**<br>*Br J Haematol 2007*, 139(4):578-89	2
Armstrong SA, Staunton JE, Korsmeyer SJ et al.<br>***MLL* translocations specify a distinct gene expression profile that distinguishes a unique leukemia.**<br>*Nat Genet 2002*, 30(1):41-7	2
Ferrando AA, Armstrong SA, Look AT et al.<br>**Gene expression signatures in *MLL*-rearranged T-lineage and B-precursor acute leukemias: dominance of *HOX* dysregulation.**<br>*Blood 2003*, 102(1):262-8	1

Table S2 - Differential expression between AML and ALL: Ranking of gene signatures

The full ranking of all signatures is shown based on the van Delft et al. dataset analyzing differential expression between AML and ALL. The third column indicates the type of the signature (D $\widehat{=}$ *Diagnostic* signature, P $\widehat{=}$ *Prognostic* signature, O $\widehat{=}$ *Other* signature). The column *Size* gives the number of probe sets in the query dataset that correspond to each gene signature. $\tilde{S}$ is the standardized score statistic of the global test that is used for ranking gene signatures in cases where a reliable asymptotic p-value cannot be guaranteed (unadjusted $p < 10^{-12}$). The last two columns show the Family Wise Error Rate as well as the associated taxonomy terms.

Rank	Gene signature	Type	Size	Array	$\tilde{S}$	FWER	Taxonomy terms
1	van Delft et al., Br J Haematol, 2005, ALL, AML	D	119	Affymetrix HG-U133A	46.40	$< 1.22\ 10^{-10}$	ALL, AML
2	Golub et al., Science, 1999, ALL, AML	D	101	Affymetrix HuGeneFl	40.93	$< 1.22\ 10^{-10}$	ALL, AML
3	van Delft et al., Br J Haematol, 2005, ALL, AML	D	126	Affymetrix HG-U133A	38.12	$< 1.22\ 10^{-10}$	ALL, AML
4	Valk et al., N Engl J Med, 2004, AML, mostly EVI1	D	59	Affymetrix HG-U133A	35.85	$< 1.22\ 10^{-10}$	
5	van Delft et al., Br J Haematol, 2005, AML, RUNX1-ETO	D	68	Affymetrix HG-U133A	34.98	$< 1.22\ 10^{-10}$	Core binding factor aberration, t(8;21), Chromosomal aberration
6	Lindvall et al., Haematologica, 2004, AML, normal vs complex karyotype	D	101	custom array	34.30	$< 1.22\ 10^{-10}$	Normal karyotype, Chromosomal aberration, Complex karyotype
7	Yagi et al., Blood, 2003, AML, good vs. poor prognosis	P	65	Affymetrix HG-U95Av2	34.22	$< 1.22\ 10^{-10}$	Leukemia, AML
8	van Delft et al., Br J Haematol, 2005, AML, t(11q23)/MLL	D	65	Affymetrix HG-U133A	34.07	$< 1.22\ 10^{-10}$	Chromosomal aberration, t(11q23)/*MLL*
9	Stirewalt et al., Genes Chromosomes Cancer, 2008, AML	D	511	Affymetrix HG-U133A	33.60	$< 1.22\ 10^{-10}$	Leukemia, Healthy, AML
10	Ross et al., Blood, 2004, AML, t(15;17)	D	159	Affymetrix HG-U133A	32.75	$< 1.22\ 10^{-10}$	t(15;17), Chromosomal aberration
11	Verhaak et al., Blood, 2005, NPM1	D	661	Affymetrix HG-U133A	32.53	$< 1.22\ 10^{-10}$	*NPM1* mutated
12	Verhaak et al., Haematologica, 2009, AML, 3q abnormality	D	73	Affymetrix HG-U133Plus2	31.60	$< 1.22\ 10^{-10}$	3q abnormality, Chromosomal aberration
13	Bullinger et al., N Engl J Med, 2004, AML, good vs. poor prognosis	P	185	custom array	31.57	$< 1.22\ 10^{-10}$	Leukemia, AML

14	Mullighan et al., Leukemia, 2007, AML, t(11q23)/MLL	D	823	Affymetrix HG-U133A	31.06	$< 1.22\ 10^{-10}$	Chromosomal aberration, t(11q23)/*MLL*
15	Verhaak et al., Haematologica, 2009, AML, FLT3-TKD	D	519	Affymetrix HG-U133Plus2	30.91	$< 1.22\ 10^{-10}$	*FLT3*-TKD, *FLT3*
16	Marcucci et al., J Clin Oncol, 2008, AML, normal karyotype, CEBPA	D	541	Affymetrix HG-U133Plus2	30.69	$< 1.22\ 10^{-10}$	*CEBPA*
17	Debernardi et al., Genes Chromosomes Cancer, 2003, AML, different karyotypes	D	221	Affymetrix HG-U95Av2	30.59	$< 1.22\ 10^{-10}$	
18	Valk et al., N Engl J Med, 2004, AML, classification of prognostically important subtypes	D	244	Affymetrix HG-U133A	30.26	$< 1.22\ 10^{-10}$	
19	Mullighan et al., Leukemia, 2007, AML, t(11q23)/MLL	D	842	Affymetrix HG-U133A	29.95	$< 1.22\ 10^{-10}$	Chromosomal aberration, t(11q23)/*MLL*
20	Langer et al., Blood, 2008, CN-AML, BAALC	D	279	Affymetrix HG-U133Plus2	29.92	$< 1.22\ 10^{-10}$	
21	Mullighan et al., Leukemia, 2007, AML, NPM1	D	441	Affymetrix HG-U133A	29.07	$< 1.22\ 10^{-10}$	*NPM1* mutated
22	Valk et al., N Engl J Med, 2004, AML, cluster without predominant characteristics	D	73	Affymetrix HG-U133A	29.01	$< 1.22\ 10^{-10}$	
23	Verhaak et al., Haematologica, 2009, AML, FLT3-ITD and/or FLT3-TKD	D	353	Affymetrix HG-U133Plus2	28.71	$< 1.22\ 10^{-10}$	*FLT3*
24	Mullighan et al., Leukemia, 2007, AML, NPM1	D	917	Affymetrix HG-U133A	27.78	$< 1.22\ 10^{-10}$	*NPM1* mutated
25	Mullighan et al., Leukemia, 2007, AML, NPM1	D	822	Affymetrix HG-U133A	26.98	$< 1.22\ 10^{-10}$	*NPM1* mutated
26	Wouters et al., Blood, 2007, AML, CEBPA	D	474	Affymetrix HG-U133A	25.82	$< 1.22\ 10^{-10}$	*CEBPA*
27	Verhaak et al., Haematologica, 2009, AML, del(5q)	D	59	Affymetrix HG-U133Plus2	28.41	$1.22\ 10^{-10}$	Chromosomal aberration, del(5q)
28	Alcalay et al., Blood, 2005, AML, NPM1, ANOVA	D	575	Affymetrix HG-U133A	26.31	$1.34\ 10^{-10}$	*NPM1* mutated
29	Ross et al., Blood, 2004, AML, t(11q23)/MLL	D	137	Affymetrix HG-U133A	30.15	$1.55\ 10^{-10}$	Chromosomal aberration, t(11q23)/*MLL*
30	van Delft et al., Br J Haematol, 2005, AML, RUNX1-ETO	D	52	Affymetrix HG-U133A	33.10	$1.55\ 10^{-10}$	Core binding factor aberration, t(8;21), Chromosomal aberration

31	Ferrando et al., Blood, 2003, T-ALL, t(11q23)/MLL	D	343	Affymetrix HuGeneFl	28.48	$1.78\ 10^{-10}$	Chromosomal aberration, t(11q23)/*MLL*
32	Ross et al., Blood, 2003, B-ALL, t(11q23)/MLL	D	112	Affymetrix HG-U133 Set	30.60	$2.91\ 10^{-10}$	
33	Alcalay et al., Blood, 2005, AML, NPM1, SAM	D	216	Affymetrix HG-U133A	25.91	$3.52\ 10^{-10}$	*NPM1* mutated
34	Armstrong et al., Nat Genet, 2002, ALL, t(11q23)/MLL	D	178	Affymetrix HG-U95Av2	31.50	$4.14\ 10^{-10}$	Chromosomal aberration, t(11q23)/*MLL*
35	Hofmann et al., Lancet, 2002, Ph+ ALL and prediction of STI571 resistance	O	148	Affymetrix HuGeneFl	25.51	$9.82\ 10^{-10}$	Leukemia, Chromosomal aberration, t9-22, ALL
36	Verhaak et al., Haematologica, 2009, AML, FLT3-ITD without NPM1	D	189	Affymetrix HG-U133Plus2	26.96	$1.03\ 10^{-9}$	*FLT3*, *FLT3*-ITD
37	Armstrong et al., Nat Genet, 2002, AML, ALL with and without t(11q23)/MLL	D	73	Affymetrix HG-U95Av2	31.24	$1.03\ 10^{-9}$	
38	Lacayo et al., Blood, 2004, FLT3	P	123	custom array	22.48	$1.15\ 10^{-9}$	Leukemia, *FLT3*, *FLT3*-ITD, AML
39	Valk et al., N Engl J Med, 2004, AML, mostly CEBPA	D	68	Affymetrix HG-U133A	23.02	$1.27\ 10^{-9}$	*CEBPA*
40	Cheok et al., Nat Genet, 2003, ALL, discrimination of treatments by gene expression	O	168	Affymetrix HG-U95Av2	25.46	$1.53\ 10^{-9}$	Leukemia, ALL
41	Verhaak et al., Haematologica, 2009, AML, KRAS	D	163	Affymetrix HG-U133Plus2	26.20	$1.56\ 10^{-9}$	*KRAS* mutated
42	Ross et al., Blood, 2003, B-ALL, t(1;19)	D	109	Affymetrix HG-U133 Set	28.24	$1.67\ 10^{-9}$	
43	Ross et al., Blood, 2004, AML, CBF	D	152	Affymetrix HG-U133A	26.00	$1.68\ 10^{-9}$	Core binding factor aberration, Chromosomal aberration
44	Valk et al., N Engl J Med, 2004, AML, mostly FLT3-ITD	D	59	Affymetrix HG-U133A	21.61	$1.89\ 10^{-9}$	
45	Pellagatti et al., Blood, 2006, MDS vs. healthy	D	19	Affymetrix HG-U133Plus2	29.62	$2.12\ 10^{-9}$	Leukemia, MDS, Healthy
46	Virtaneva et al., Proc Natl Acad Sci U S A, 2001, AML	D	99	Affymetrix HuGeneFl	21.12	$2.52\ 10^{-9}$	Normal karyotype, Chromosomal aberration, Trisomy 8

47	Boultwood et al., Br J Haematol, 2007, MDS 5q- vs. RA normal karyotype	D	91	Affymetrix HG-U133Plus2	18.55	$2.92\ 10^{-9}$	Normal karyotype, Chromosomal aberration, del(5q)
48	Yeoh et al., Cancer Cell, 2002, ALL, t(1;19)	D	66	Affymetrix HG-U95Av2	22.29	$3.20\ 10^{-9}$	
49	Valk et al., N Engl J Med, 2004, AML, t(15;17)	D	60	Affymetrix HG-U133A	20.77	$4.57\ 10^{-9}$	t(15;17), Chromosomal aberration
50	Kohlmann et al., Genes Chromosomes Cancer, 2003, ALL	D	31	Affymetrix HG-U95Av2 + HG-U133A	27.29	$6.26\ 10^{-9}$	
51	Ferrando et al., Cancer Cell, 2002, T-ALL, HOX11	O	40	Affymetrix HuGeneFl	26.01	$6.26\ 10^{-9}$	Leukemia, ALL, T-ALL
52	Verhaak et al., Haematologica, 2009, AML, NRAS	D	216	Affymetrix HG-U133Plus2	26.51	$6.26\ 10^{-9}$	*NRAS* mutated
53	Boultwood et al., Br J Haematol, 2007, MDS 5q- vs. healthy	D	74	Affymetrix HG-U133Plus2	28.00	$9.00\ 10^{-9}$	
54	Metzeler et al., Blood, 2008, CN-AML, Survival	P	107	Affymetrix HG-U133 Set	25.29	$9.00\ 10^{-9}$	Leukemia, Normal karyotype, AML
55	Wouters et al., Blood, 2009, AML, CEBPA	D	19	Affymetrix HG-U133Plus2	18.67	$9.19\ 10^{-9}$	*CEBPA*
56	Whitman et al., Blood, 2008, AML, FLT3-TKD vs. FLT3 wild type	D	371	Affymetrix HG-U133Plus2	23.14	$9.71\ 10^{-9}$	*FLT3*-TKD, *FLT3*
57	Stirewalt et al., Genes Chromosomes Cancer, 2008, AML	D	38	Affymetrix HG-U133A	18.69	$1.05\ 10^{-8}$	Leukemia, Healthy, AML
58	Valk et al., N Engl J Med, 2004, AML, mostly inv(16)	D	48	Affymetrix HG-U133A	18.67	$1.60\ 10^{-8}$	Core binding factor aberration, inv(16), Chromosomal aberration
59	Verhaak et al., Haematologica, 2009, AML, FLT3-ITD	D	56	Affymetrix HG-U133Plus2	22.23	$1.69\ 10^{-8}$	*FLT3*, *FLT3*-ITD
60	Ross et al., Blood, 2004, Leukemia, t(11q23)/MLL	D	146	Affymetrix HG-U133A	23.03	$2.15\ 10^{-8}$	Chromosomal aberration, t(11q23)/*MLL*
61	Verhaak et al., Haematologica, 2009, AML, del(7q)	D	90	Affymetrix HG-U133Plus2	18.78	$2.19\ 10^{-8}$	del(7q), Chromosomal aberration
62	van Delft et al., Br J Haematol, 2005, ALL, pre-B, hyperdiploid	D	67	Affymetrix HG-U133A	26.60	$2.56\ 10^{-8}$	Hyperdiploid > 50 chromosomes, Hyperdiploid, Chromosomal aberration

63	Verhaak et al., Haematologica, 2009, AML, t(11q23)/MLL	D	27	Affymetrix HG-U133Plus2	21.02	$2.72\ 10^{-8}$	Chromosomal aberration, t(11q23)/*MLL*
64	Yeoh et al., Cancer Cell, 2002, ALL, novel ALL subgroup with common gene expression	D	53	Affymetrix HG-U95Av2	19.33	$4.49\ 10^{-8}$	B-ALL
65	Ross et al., Blood, 2003, ALL classification	D	358	Affymetrix HG-U133 Set	24.13	$4.49\ 10^{-8}$	
66	Virtaneva et al., Proc Natl Acad Sci U S A, 2001, AML	D	127	Affymetrix HuGeneFl	22.87	$4.53\ 10^{-8}$	
67	Cheok et al., Nat Genet, 2003, ALL, discrimination of treatments by gene expression	O	78	Affymetrix HG-U95Av2	19.74	$5.09\ 10^{-8}$	Leukemia, ALL
68	Ross et al., Blood, 2003, B-ALL, t(9;22)	D	111	Affymetrix HG-U133 Set	17.52	$5.35\ 10^{-8}$	
69	Marcucci et al., J Clin Oncol, 2005, AML, ERG	O	17	Affymetrix HG-U133Plus2	22.82	$6.08\ 10^{-8}$	Leukemia, Normal karyotype, AML
70	Yeoh et al., Cancer Cell, 2002, ALL, t(11q23)/MLL	D	76	Affymetrix HG-U95Av2	24.43	$6.34\ 10^{-8}$	
71	Cheok et al., Nat Genet, 2003, ALL, discrimination of treatments by gene expression	O	84	Affymetrix HG-U95Av2	18.30	$6.72\ 10^{-8}$	Leukemia, ALL
72	Ross et al., Blood, 2004, AML, outcome prediction	P	92	Affymetrix HG-U133A	18.97	$6.72\ 10^{-8}$	Leukemia, AML
73	Cheok et al., Nat Genet, 2003, ALL, discrimination of treatments by gene expression	O	242	Affymetrix HG-U95Av2	20.79	$7.33\ 10^{-8}$	Leukemia, ALL
74	Hofmann et al., Lancet, 2002, Ph+ ALL and development of STI571 resistance	O	101	Affymetrix HuGeneFl	16.56	$9.33\ 10^{-8}$	Leukemia, Chromosomal aberration, t9-22, ALL
75	Ross et al., Blood, 2004, AML, FAB M7	D	147	Affymetrix HG-U133A	23.82	$9.59\ 10^{-8}$	
76	Verhaak et al., Haematologica, 2009, AML, NPM1 without FLT3-ITD	D	81	Affymetrix HG-U133Plus2	19.91	$1.18\ 10^{-7}$	*NPM1* mutated
77	Ross et al., Blood, 2004, AML, t(8;21)	D	125	Affymetrix HG-U133A	21.76	$1.52\ 10^{-7}$	Core binding factor aberration, t(8;21), Chromosomal aberration
78	Ross et al., Blood, 2004, AML, inv(16)	D	91	Affymetrix HG-U133A	20.03	$1.94\ 10^{-7}$	Core binding factor aberration, inv(16), Chromosomal aberration
79	van Delft et al., Br J Haematol, 2005, AML, t(11q23)/MLL	D	54	Affymetrix HG-U133A	22.40	$2.20\ 10^{-7}$	Chromosomal aberration, t(11q23)/*MLL*

80	Virtaneva et al., Proc Natl Acad Sci U S A, 2001, AML	D	130	Affymetrix HuGeneFl	20.55	$2.55\ 10^{-7}$	
81	Valk et al., N Engl J Med, 2004, AML, mostly t(11q23)/MLL	D	64	Affymetrix HG-U133A	20.59	$2.55\ 10^{-7}$	
82	Kohlmann et al., Leukemia, 2004, ALL	D	767	Affymetrix HG-U133A	20.56	$2.67\ 10^{-7}$	
83	Yeoh et al., Cancer Cell, 2002, ALL, hyper-diploid ¿50	D	70	Affymetrix HG-U95Av2	18.78	$3.70\ 10^{-7}$	
84	Valk et al., N Engl J Med, 2004, AML, mostly FAB M4 and M5	D	69	Affymetrix HG-U133A	21.41	$4.95\ 10^{-7}$	
85	Cheok et al., Nat Genet, 2003, ALL, discrimination of treatments by gene expression	O	98	Affymetrix HG-U95Av2	19.28	$5.05\ 10^{-7}$	Leukemia, ALL
86	Lacayo et al., Blood, 2004, FLT3-mutated vs. FLT3 wild type	D	40	custom array	17.49	$5.76\ 10^{-7}$	*FLT3*
87	Valk et al., N Engl J Med, 2004, AML, CEBPA	D	74	Affymetrix HG-U133A	17.37	$6.20\ 10^{-7}$	*CEBPA*
88	Verhaak et al., Haematologica, 2009, AML, NPM1 and FLT3-ITD	D	37	Affymetrix HG-U133Plus2	18.48	$6.33\ 10^{-7}$	
89	Bhojwani et al., J Clin Oncol, 2008, ALL, long-term outcome	P	75	Affymetrix HG-U133Plus2	20.24	$7.43\ 10^{-7}$	Leukemia, B-ALL, pre-B-ALL, ALL
90	Virtaneva et al., Proc Natl Acad Sci U S A, 2001, AML	D	116	Affymetrix HuGeneFl	20.04	$7.43\ 10^{-7}$	Leukemia, Healthy, AML
91	Ferrando et al., Cancer Cell, 2002, T-ALL, TAL1	O	38	Affymetrix HuGeneFl	16.74	$7.87\ 10^{-7}$	Leukemia, ALL, T-ALL
92	Schoch et al., Proc Natl Acad Sci U S A, 2002, AML	D	19	Affymetrix HG-U95Av2	18.04	$1.48\ 10^{-6}$	
93	Lacayo et al., Blood, 2004, FLT3-mutated	P	155	custom array	14.67	$1.57\ 10^{-6}$	Leukemia, *FLT3*, AML
94	van Delft et al., Br J Haematol, 2005, ALL, pre-B, ETV6-RUNX1	D	67	Affymetrix HG-U133A	19.99	$1.91\ 10^{-6}$	t(12;21), Chromosomal aberration
95	Valk et al., N Engl J Med, 2004, AML, mostly t(11q23)/MLL	D	54	Affymetrix HG-U133A	17.67	$2.80\ 10^{-6}$	
96	Verhaak et al., Haematologica, 2009, AML, t(8;21)	D	77	Affymetrix HG-U133Plus2	17.09	$3.58\ 10^{-6}$	Core binding factor aberration, t(8;21), Chromosomal aberration
97	van Delft et al., Br J Haematol, 2005, AML, FAB M7	D	65	Affymetrix HG-U133A	18.49	$5.17\ 10^{-6}$	

98	van Delft et al., Br J Haematol, 2005, ALL, pre-B, RUNX1 amplification	D	87	Affymetrix HG-U133A	16.75	$5.50\ 10^{-6}$	
99	Wouters et al., Blood, 2009, AML, CEBPA double mutation	D	19	Affymetrix HG-U133Plus2	13.31	$5.82\ 10^{-6}$	*CEBPA* double mutant, *CEBPA*
100	Yeoh et al., Cancer Cell, 2002, ALL, T- vs. B-lineage	D	68	Affymetrix HG-U95Av2	18.18	$6.74\ 10^{-6}$	B-ALL, T-ALL
101	Vey et al., Oncogene, 2004, AML, t(15;17)	D	14	custom array	12.13	$6.99\ 10^{-6}$	t(15;17), Chromosomal aberration
102	van Delft et al., Br J Haematol, 2005, AML, FAB M7	D	82	Affymetrix HG-U133A	16.56	$7.65\ 10^{-6}$	
103	Verhaak et al., Haematologica, 2009, AML, CEBPA	D	8	Affymetrix HG-U133Plus2	13.11	$8.88\ 10^{-6}$	*CEBPA*
104	Vey et al., Oncogene, 2004, AML, t(8;21)	D	13	custom array	16.11	$1.07\ 10^{-5}$	Core binding factor aberration, t(8;21), Chromosomal aberration
105	Ross et al., Blood, 2003, B-ALL vs. T-ALL	D	130	Affymetrix HG-U133 Set	16.09	$1.52\ 10^{-5}$	B-ALL, T-ALL
106	Kohlmann et al., Genes Chromosomes Cancer, 2003, AML	D	44	Affymetrix HG-U95Av2 + HG-U133A	15.31	$1.77\ 10^{-5}$	
107	Verhaak et al., Haematologica, 2009, AML, NPM1	D	47	Affymetrix HG-U133Plus2	15.02	$2.10\ 10^{-5}$	*NPM1* mutated
108	Valk et al., N Engl J Med, 2004, AML, t(8;21)	D	56	Affymetrix HG-U133A	14.49	$2.26\ 10^{-5}$	Core binding factor aberration, t(8;21), Chromosomal aberration
109	Vey et al., Oncogene, 2004, AML, t(11;19)	D	10	custom array	14.42	$2.26\ 10^{-5}$	t(11;19), Chromosomal aberration
110	Bhojwani et al., J Clin Oncol, 2008, ALL, therapy response	O	26	Affymetrix HG-U133Plus2	15.54	$2.42\ 10^{-5}$	Leukemia, B-ALL, pre-B-ALL, ALL
111	Yeoh et al., Cancer Cell, 2002, ALL, t(12;21)	D	55	Affymetrix HG-U95Av2	15.60	$2.91\ 10^{-5}$	
112	van Delft et al., Br J Haematol, 2005, ALL, pre-B, hyperdiploid	D	53	Affymetrix HG-U133A	15.08	$3.73\ 10^{-5}$	Hyperdiploid > 50 chromosomes, Hyperdiploid, Chromosomal aberration
113	Yeoh et al., Cancer Cell, 2002, ALL, t(9;22)	D	66	Affymetrix HG-U95Av2	11.19	$5.37\ 10^{-5}$	

114	Verhaak et al., Haematologica, 2009, AML, t(15;17)	D	11	Affymetrix HG-U133Plus2	12.58	5.92 10^{-5}	t(15;17), Chromosomal aberration
115	Cheok et al., Nat Genet, 2003, ALL, treatment induced gene expression changes	O	33	Affymetrix HG-U95Av2	12.33	6.10 10^{-5}	Leukemia, ALL
116	Ferrando et al., Cancer Cell, 2002, T-ALL, LYL1	O	29	Affymetrix HuGeneFl	11.25	8.51 10^{-5}	Leukemia, ALL, T-ALL
117	van Delft et al., Br J Haematol, 2005, ALL, pre-B, E2A-PBX1	D	109	Affymetrix HG-U133A	11.16	1.28 10^{-4}	Chromosomal aberration, t(1;19)
118	Pellagatti et al., Blood, 2006, MDS, 5q- vs. other	D	53	Affymetrix HG-U133Plus2	11.88	1.77 10^{-4}	Chromosomal aberration, del(5q)
119	Schoch et al., Proc Natl Acad Sci U S A, 2002, AML	D	53	Affymetrix HG-U95Av2	11.42	1.79 10^{-4}	
120	Valk et al., N Engl J Med, 2004, AML, mostly FLT3-ITD	D	59	Affymetrix HG-U133A	11.78	1.93 10^{-4}	
121	Cheok et al., Nat Genet, 2003, ALL, discrimination of treatments by gene expression	O	85	Affymetrix HG-U95Av2	11.18	2.49 10^{-4}	Leukemia, ALL
122	van Delft et al., Br J Haematol, 2005, ALL, pre-B, RUNX1 amplification	D	57	Affymetrix HG-U133A	10.57	3.09 10^{-4}	
123	van Delft et al., Br J Haematol, 2005, ALL, pre-B, ETV6-RUNX1	D	45	Affymetrix HG-U133A	11.53	4.50 10^{-4}	t(12;21), Chromosomal aberration
124	Ross et al., Blood, 2003, B-ALL, t(12;21)	D	98	Affymetrix HG-U133 Set	11.23	4.63 10^{-4}	
125	Ross et al., Blood, 2003, B-ALL, hyperdiploid	D	133	Affymetrix HG-U133 Set	9.83	8.18 10^{-4}	
126	Verhaak et al., Haematologica, 2009, AML, t(6;9)	D	15	Affymetrix HG-U133Plus2	7.25	3.31 10^{-3}	t(6;9), Chromosomal aberration
127	Valk et al., N Engl J Med, 2004, AML, mostly FLT3-ITD	D	57	Affymetrix HG-U133A	8.14	3.31 10^{-3}	
128	Cheok et al., Nat Genet, 2003, ALL, discrimination of treatments by gene expression	O	41	Affymetrix HG-U95Av2	7.55	6.15 10^{-3}	Leukemia, ALL
129	Valk et al., N Engl J Med, 2004, AML, cluster without predominant characteristics	D	69	Affymetrix HG-U133A	7.38	6.15 10^{-3}	
130	van Delft et al., Br J Haematol, 2005, ALL, T-ALL	D	72	Affymetrix HG-U133A	6.38	1.21 10^{-2}	B-ALL, pre-B-ALL, T-ALL
131	Vey et al., Oncogene, 2004, AML, normal karyotype, good vs. poor outcome	P	44	custom array	5.79	1.49 10^{-2}	Leukemia, Normal karyotype, AML

132	Vey et al., Oncogene, 2004, AML, normal karyotype	D	11	custom array	5.58	1.92 10^{-2}	Normal karyotype
133	van Delft et al., Br J Haematol, 2005, ALL, pre-B, E2A-PBX1	D	49	Affymetrix HG-U133A	4.96	1.92 10^{-2}	Chromosomal aberration, t(1;19)
134	Valk et al., N Engl J Med, 2004, AML, cluster without predominant characteristics	D	68	Affymetrix HG-U133A	5.05	1.92 10^{-2}	
135	van Delft et al., Br J Haematol, 2005, ALL, T-ALL	D	50	Affymetrix HG-U133A	4.02	3.82 10^{-2}	B-ALL, pre-B-ALL, T-ALL
136	Cheok et al., Nat Genet, 2003, ALL, treatment induced gene expression changes	O	39	Affymetrix HG-U95Av2	3.28	3.86 10^{-2}	Leukemia, ALL
137	Ebert et al., PLoS Med, 2008, MDS, response to lenalidomide	O	93	Affymetrix HG-U133Plus2	3.57	3.86 10^{-2}	Leukemia, MDS
138	Cheok et al., Nat Genet, 2003, ALL, treatment induced gene expression changes	O	22	Affymetrix HG-U95Av2	1.19	1.03 10^{-1}	Leukemia, ALL

Table S3 - Differential expression between AML and ALL: Ranking of taxonomy terms

Rank	unadjusted p-value	Taxonomy term	Number of signatures	Number of articles
1	0.002	ALL	3	2
2	0.005	AML	6	4
3	0.013	t11q23	9	6
4	0.029	NPMmut	8	4
5	0.061	ComplexKaryotype	1	1
6	0.082	abn3q	1	1
7	0.102	FLT3mut	6	3
8	0.134	FLT3-TKD	2	2
9	0.205	Leukemia	4	3
10	0.205	Healthy	4	3
11	0.268	KRASmut	1	1
12	0.273	FLT3-ITD	2	1
13	0.310	MDS	1	1
14	0.321	Trisomy8	1	1
15	0.333	NormalKaryotype	4	4
16	0.346	CEBPAmut	7	5
17	0.379	NRASmut	1	1
18	0.400	ChromosomalAberration	37	12
19	0.471	del5q	3	3
20	0.475	del7q	1	1
21	0.500	CoreBindingFactorAberration	9	5
22	0.531	inv16	2	2
23	0.547	t15-17	4	4
24	0.579	t8-21	6	5
25	0.789	HyperdiploidOver50	2	1
26	0.789	Hyperdiploid	2	1
27	0.789	CEBPA-double-mut	1	1
28	0.867	t11-19	1	1
29	0.928	t12-21	2	1
30	0.935	t6-9	1	1
31	0.981	t1-19	2	1
32	0.990	B-ALL	5	3
33	0.991	pre-B-ALL	2	1
34	0.995	T-ALL	4	3

Table S4 - *NPM1* mutation in AML with normal karyotype: Ranking of gene signatures

The full ranking of all signatures is shown based on the normal karyotype AML dataset analyzing differential expression between *NPM1*-mutated and *NPM1* wild type cases. The third column indicates the type of the signature (D $\widehat{=}$ *Diagnostic* signature, P $\widehat{=}$ *Prognostic* signature, O $\widehat{=}$ *Other* signature). The column *Size* gives the number of probe sets in the query dataset that correspond to each gene signature. $\tilde{S}$ is the standardized score statistic of the global test that is used for ranking gene signatures in cases where a reliable asymptotic p-value cannot be guaranteed (unadjusted $p < 10^{-12}$). The last two columns show the Family Wise Error Rate as well as the associated taxonomy terms.

Rank	Gene signature	Type	Size	Array	$\tilde{S}$	FWER	Taxonomy terms
1	Verhaak et al., Haematologica, 2009, AML, NPM1	D	89	Affymetrix HG-U133Plus2	115.23	$< 8.77\ 10^{-11}$	*NPM1* mutated
2	Verhaak et al., Haematologica, 2009, AML, NPM1 and FLT3-ITD	D	71	Affymetrix HG-U133Plus2	99.51	$< 8.77\ 10^{-11}$	
3	Verhaak et al., Haematologica, 2009, AML, NPM1 without FLT3-ITD	D	138	Affymetrix HG-U133Plus2	95.01	$< 8.77\ 10^{-11}$	*NPM1* mutated
4	Verhaak et al., Haematologica, 2009, AML, FLT3-ITD and/or FLT3-TKD	D	649	Affymetrix HG-U133Plus2	84.54	$< 8.77\ 10^{-11}$	*FLT3*
5	Alcalay et al., Blood, 2005, AML, NPM1, SAM	D	277	Affymetrix HG-U133A	82.92	$< 8.77\ 10^{-11}$	*NPM1* mutated
6	Verhaak et al., Haematologica, 2009, AML, FLT3-ITD	D	97	Affymetrix HG-U133Plus2	80.64	$< 8.77\ 10^{-11}$	*FLT3*, *FLT3*-ITD
7	Alcalay et al., Blood, 2005, AML, NPM1, ANOVA	D	754	Affymetrix HG-U133A	74.71	$< 8.77\ 10^{-11}$	*NPM1* mutated
8	Valk et al., N Engl J Med, 2004, AML, classification of prognostically important subtypes	D	304	Affymetrix HG-U133A	70.86	$< 8.77\ 10^{-11}$	
9	Ross et al., Blood, 2004, Leukemia, t(11q23)/MLL	D	185	Affymetrix HG-U133A	70.22	$< 8.77\ 10^{-11}$	Chromosomal aberration, t(11q23)/*MLL*
10	Mullighan et al., Leukemia, 2007, AML, NPM1	D	557	Affymetrix HG-U133A	69.13	$< 8.77\ 10^{-11}$	*NPM1* mutated
11	Mullighan et al., Leukemia, 2007, AML, NPM1	D	1141	Affymetrix HG-U133A	68.34	$< 8.77\ 10^{-11}$	*NPM1* mutated
12	Verhaak et al., Haematologica, 2009, AML, del(7q)	D	166	Affymetrix HG-U133Plus2	67.34	$< 8.77\ 10^{-11}$	del(7q), Chromosomal aberration
13	Mullighan et al., Leukemia, 2007, AML, NPM1	D	1013	Affymetrix HG-U133A	65.35	$< 8.77\ 10^{-11}$	*NPM1* mutated

14	Verhaak et al., Haematologica, 2009, AML, t(15;17)	D	19	Affymetrix HG-U133Plus2	63.31	$< 8.77\ 10^{-11}$	t(15;17), Chromosomal aberration
15	Marcucci et al., J Clin Oncol, 2008, AML, normal karyotype, CEBPA	D	845	Affymetrix HG-U133Plus2	63.26	$< 8.77\ 10^{-11}$	*CEBPA*
16	Stirewalt et al., Genes Chromosomes Cancer, 2008, AML	D	48	Affymetrix HG-U133A	62.27	$< 8.77\ 10^{-11}$	Leukemia, Healthy, AML
17	Valk et al., N Engl J Med, 2004, AML, CEBPA	D	93	Affymetrix HG-U133A	61.51	$< 8.77\ 10^{-11}$	*CEBPA*
18	Ross et al., Blood, 2003, B-ALL, t(11q23)/MLL	D	183	Affymetrix HG-U133 Set	61.37	$< 8.77\ 10^{-11}$	
19	van Delft et al., Br J Haematol, 2005, AML, t(11q23)/MLL	D	76	Affymetrix HG-U133A	60.52	$< 8.77\ 10^{-11}$	Chromosomal aberration, t(11q23)/*MLL*
20	Valk et al., N Engl J Med, 2004, AML, cluster without predominant characteristics	D	100	Affymetrix HG-U133A	60.39	$< 8.77\ 10^{-11}$	
21	Verhaak et al., Blood, 2005, NPM1	D	840	Affymetrix HG-U133A	57.67	$< 8.77\ 10^{-11}$	*NPM1* mutated
22	Langer et al., Blood, 2008, CN-AML, BAALC	D	544	Affymetrix HG-U133Plus2	56.81	$< 8.77\ 10^{-11}$	
23	van Delft et al., Br J Haematol, 2005, AML, t(11q23)/MLL	D	89	Affymetrix HG-U133A	56.79	$< 8.77\ 10^{-11}$	Chromosomal aberration, t(11q23)/*MLL*
24	Armstrong et al., Nat Genet, 2002, ALL, t(11q23)/MLL	D	241	Affymetrix HG-U95Av2	52.72	$< 8.77\ 10^{-11}$	Chromosomal aberration, t(11q23)/*MLL*
25	Valk et al., N Engl J Med, 2004, AML, mostly EVI1	D	72	Affymetrix HG-U133A	51.43	$< 8.77\ 10^{-11}$	
26	Ross et al., Blood, 2004, AML, t(8;21)	D	152	Affymetrix HG-U133A	51.13	$< 8.77\ 10^{-11}$	Core binding factor aberration, t(8;21), Chromosomal aberration
27	Lindvall et al., Haematologica, 2004, AML, normal vs complex karyotype	D	167	custom array	50.89	$< 8.77\ 10^{-11}$	Normal karyotype, Chromosomal aberration, Complex karyotype
28	Ross et al., Blood, 2004, AML, t(15;17)	D	204	Affymetrix HG-U133A	49.48	$< 8.77\ 10^{-11}$	t(15;17), Chromosomal aberration
29	Valk et al., N Engl J Med, 2004, AML, t(8;21)	D	65	Affymetrix HG-U133A	47.36	$< 8.77\ 10^{-11}$	Core binding factor aberration, t(8;21), Chromosomal aberration

30	Valk et al., N Engl J Med, 2004, AML, mostly inv(16)	D	71	Affymetrix HG-U133A	47.32	$< 8.77\ 10^{-11}$	Core binding factor aberration, inv(16), Chromosomal aberration
31	Valk et al., N Engl J Med, 2004, AML, mostly FLT3-ITD	D	78	Affymetrix HG-U133A	45.60	$< 8.77\ 10^{-11}$	
32	Golub et al., Science, 1999, ALL, AML	D	119	Affymetrix HuGeneFl	42.68	$< 8.77\ 10^{-11}$	ALL, AML
33	Valk et al., N Engl J Med, 2004, AML, mostly CEBPA	D	89	Affymetrix HG-U133A	41.78	$< 8.77\ 10^{-11}$	*CEBPA*
34	Stirewalt et al., Genes Chromosomes Cancer, 2008, AML	D	641	Affymetrix HG-U133A	40.32	$< 8.77\ 10^{-11}$	Leukemia, Healthy, AML
35	Verhaak et al., Haematologica, 2009, AML, t(8;21)	D	120	Affymetrix HG-U133Plus2	40.25	$< 8.77\ 10^{-11}$	Core binding factor aberration, t(8;21), Chromosomal aberration
36	Ross et al., Blood, 2004, AML, t(11q23)/MLL	D	176	Affymetrix HG-U133A	39.66	$< 8.77\ 10^{-11}$	Chromosomal aberration, t(11q23)/*MLL*
37	Mullighan et al., Leukemia, 2007, AML, t(11q23)/MLL	D	1062	Affymetrix HG-U133A	39.46	$< 8.77\ 10^{-11}$	Chromosomal aberration, t(11q23)/*MLL*
38	Mullighan et al., Leukemia, 2007, AML, t(11q23)/MLL	D	1050	Affymetrix HG-U133A	39.40	$< 8.77\ 10^{-11}$	Chromosomal aberration, t(11q23)/*MLL*
39	Ross et al., Blood, 2004, AML, CBF	D	195	Affymetrix HG-U133A	38.82	$< 8.77\ 10^{-11}$	Core binding factor aberration, Chromosomal aberration
40	Debernardi et al., Genes Chromosomes Cancer, 2003, AML, different karyotypes	D	276	Affymetrix HG-U95Av2	38.15	$< 8.77\ 10^{-11}$	
41	Valk et al., N Engl J Med, 2004, AML, mostly FLT3-ITD	D	81	Affymetrix HG-U133A	38.10	$< 8.77\ 10^{-11}$	
42	Bullinger et al., N Engl J Med, 2004, AML, good vs. poor prognosis	P	269	custom array	37.85	$< 8.77\ 10^{-11}$	Leukemia, AML
43	Wouters et al., Blood, 2009, AML, CEBPA double mutation	D	37	Affymetrix HG-U133Plus2	36.82	$< 8.77\ 10^{-11}$	*CEBPA* double mutant, *CEBPA*
44	Verhaak et al., Haematologica, 2009, AML, 3q abnormality	D	103	Affymetrix HG-U133Plus2	36.13	$< 8.77\ 10^{-11}$	3q abnormality, Chromosomal aberration
45	van Delft et al., Br J Haematol, 2005, AML, RUNX1-ETO	D	70	Affymetrix HG-U133A	35.94	$< 8.77\ 10^{-11}$	Core binding factor aberration, t(8;21), Chromosomal aberration

46	Wouters et al., Blood, 2009, AML, CEBPA	D	38	Affymetrix HG-U133Plus2	35.54	$< 8.77\ 10^{-11}$	*CEBPA*
47	Kohlmann et al., Genes Chromosomes Cancer, 2003, AML	D	58	Affymetrix HG-U95Av2 + HG-U133A	34.92	$< 8.77\ 10^{-11}$	
48	Valk et al., N Engl J Med, 2004, AML, t(15;17)	D	77	Affymetrix HG-U133A	34.51	$< 8.77\ 10^{-11}$	t(15;17), Chromosomal aberration
49	Kohlmann et al., Leukemia, 2004, ALL	D	970	Affymetrix HG-U133A	33.60	$< 8.77\ 10^{-11}$	
50	Verhaak et al., Haematologica, 2009, AML, del(5q)	D	100	Affymetrix HG-U133Plus2	32.64	$< 8.77\ 10^{-11}$	Chromosomal aberration, del(5q)
51	Schoch et al., Proc Natl Acad Sci U S A, 2002, AML	D	26	Affymetrix HG-U95Av2	32.40	$< 8.77\ 10^{-11}$	
52	Ross et al., Blood, 2003, ALL classification	D	544	Affymetrix HG-U133 Set	31.72	$< 8.77\ 10^{-11}$	
53	Lacayo et al., Blood, 2004, FLT3-mutated	P	271	custom array	28.17	$< 8.77\ 10^{-11}$	Leukemia, *FLT3*, AML
54	Ferrando et al., Blood, 2003, T-ALL, t(11q23)/MLL	D	431	Affymetrix HuGeneFl	27.61	$< 8.77\ 10^{-11}$	Chromosomal aberration, t(11q23)/*MLL*
55	Wouters et al., Blood, 2007, AML, CEBPA	D	613	Affymetrix HG-U133A	26.50	$< 8.77\ 10^{-11}$	*CEBPA*
56	Bhojwani et al., J Clin Oncol, 2008, ALL, therapy response	O	65	Affymetrix HG-U133Plus2	26.26	$< 8.77\ 10^{-11}$	Leukemia, B-ALL, pre-B-ALL, ALL
57	Pellagatti et al., Blood, 2006, MDS vs. healthy	D	33	Affymetrix HG-U133Plus2	25.61	$< 8.77\ 10^{-11}$	Leukemia, MDS, Healthy
58	Ross et al., Blood, 2003, B-ALL, t(9;22)	D	167	Affymetrix HG-U133 Set	28.59	$8.77\ 10^{-11}$	
59	Verhaak et al., Haematologica, 2009, AML, FLT3-ITD without NPM1	D	332	Affymetrix HG-U133Plus2	25.26	$1.17\ 10^{-10}$	*FLT3*, *FLT3*-ITD
60	Whitman et al., Blood, 2008, AML, FLT3-TKD vs. FLT3 wild type	D	602	Affymetrix HG-U133Plus2	28.86	$1.41\ 10^{-10}$	*FLT3*-TKD, *FLT3*
61	Ross et al., Blood, 2003, B-ALL, t(12;21)	D	160	Affymetrix HG-U133 Set	22.54	$2.73\ 10^{-10}$	
62	Armstrong et al., Nat Genet, 2002, AML, ALL with and without t(11q23)/MLL	D	103	Affymetrix HG-U95Av2	27.58	$4.76\ 10^{-10}$	
63	Lacayo et al., Blood, 2004, FLT3-mutated vs. FLT3 wild type	D	64	custom array	20.58	$7.21\ 10^{-10}$	*FLT3*

64	Pellagatti et al., Blood, 2006, MDS, 5q- vs. other	D	89	Affymetrix HG-U133Plus2	23.32	7.32 10^{-10}	Chromosomal aberration, del(5q)
65	Verhaak et al., Haematologica, 2009, AML, FLT3-TKD	D	899	Affymetrix HG-U133Plus2	26.78	1.03 10^{-9}	*FLT3*-TKD, *FLT3*
66	Ross et al., Blood, 2003, B-ALL, t(1;19)	D	198	Affymetrix HG-U133 Set	20.79	1.53 10^{-9}	
67	Valk et al., N Engl J Med, 2004, AML, mostly t(11q23)/MLL	D	80	Affymetrix HG-U133A	20.38	4.42 10^{-9}	
68	Yeoh et al., Cancer Cell, 2002, ALL, hyper-diploid ¿50	D	81	Affymetrix HG-U95Av2	21.16	7.67 10^{-9}	
69	Ferrando et al., Cancer Cell, 2002, T-ALL, LYL1	O	33	Affymetrix HuGeneFl	22.18	9.97 10^{-9}	Leukemia, ALL, T-ALL
70	Yeoh et al., Cancer Cell, 2002, ALL, t(11q23)/MLL	D	83	Affymetrix HG-U95Av2	22.27	1.15 10^{-8}	
71	Verhaak et al., Haematologica, 2009, AML, t(6;9)	D	33	Affymetrix HG-U133Plus2	26.20	1.25 10^{-8}	t(6;9), Chromosomal aberration
72	Verhaak et al., Haematologica, 2009, AML, CEBPA	D	23	Affymetrix HG-U133Plus2	26.59	2.33 10^{-8}	*CEBPA*
73	Valk et al., N Engl J Med, 2004, AML, mostly t(11q23)/MLL	D	67	Affymetrix HG-U133A	20.60	5.02 10^{-8}	
74	Valk et al., N Engl J Med, 2004, AML, mostly FLT3-ITD	D	80	Affymetrix HG-U133A	23.36	9.44 10^{-8}	
75	Lacayo et al., Blood, 2004, FLT3	P	224	custom array	18.91	1.18 10^{-7}	Leukemia, *FLT3*, *FLT3*-ITD, AML
76	Ross et al., Blood, 2004, AML, outcome prediction	P	116	Affymetrix HG-U133A	15.13	1.54 10^{-7}	Leukemia, AML
77	van Delft et al., Br J Haematol, 2005, ALL, pre-B, ETV6-RUNX1	D	73	Affymetrix HG-U133A	20.67	3.45 10^{-7}	t(12;21), Chromosomal aberration
78	Vey et al., Oncogene, 2004, AML, normal karyotype	D	15	custom array	22.86	4.19 10^{-7}	Normal karyotype
79	Yeoh et al., Cancer Cell, 2002, ALL, novel ALL subgroup with common gene expression	D	73	Affymetrix HG-U95Av2	15.95	4.45 10^{-7}	B-ALL
80	van Delft et al., Br J Haematol, 2005, AML, FAB M7	D	70	Affymetrix HG-U133A	16.41	1.00 10^{-6}	
81	Virtaneva et al., Proc Natl Acad Sci U S A, 2001, AML	D	138	Affymetrix HuGeneFl	16.24	1.00 10^{-6}	Leukemia, Healthy, AML

82	Yeoh et al., Cancer Cell, 2002, ALL, T- vs. B-lineage	D	85	Affymetrix HG-U95Av2	16.69	$1.45\ 10^{-6}$	B-ALL, T-ALL
83	Virtaneva et al., Proc Natl Acad Sci U S A, 2001, AML	D	140	Affymetrix HuGeneFl	16.15	$1.47\ 10^{-6}$	
84	Virtaneva et al., Proc Natl Acad Sci U S A, 2001, AML	D	135	Affymetrix HuGeneFl	15.82	$1.47\ 10^{-6}$	
85	Kohlmann et al., Genes Chromosomes Cancer, 2003, ALL	D	36	Affymetrix HG-U95Av2 + HG-U133A	18.79	$1.89\ 10^{-6}$	
86	Yeoh et al., Cancer Cell, 2002, ALL, t(12;21)	D	82	Affymetrix HG-U95Av2	13.92	$1.89\ 10^{-6}$	
87	van Delft et al., Br J Haematol, 2005, AML, RUNX1-ETO	D	84	Affymetrix HG-U133A	13.93	$2.38\ 10^{-6}$	Core binding factor aberration, t(8;21), Chromosomal aberration
88	Schoch et al., Proc Natl Acad Sci U S A, 2002, AML	D	66	Affymetrix HG-U95Av2	17.12	$2.99\ 10^{-6}$	
89	Hofmann et al., Lancet, 2002, Ph+ ALL and development of STI571 resistance	O	119	Affymetrix HuGeneFl	13.39	$3.55\ 10^{-6}$	Leukemia, Chromosomal aberration, t9-22, ALL
90	Vey et al., Oncogene, 2004, AML, t(11;19)	D	13	custom array	17.18	$5.17\ 10^{-6}$	t(11;19), Chromosomal aberration
91	van Delft et al., Br J Haematol, 2005, ALL, pre-B, ETV6-RUNX1	D	97	Affymetrix HG-U133A	15.69	$5.61\ 10^{-6}$	t(12;21), Chromosomal aberration
92	Vey et al., Oncogene, 2004, AML, normal karyotype, good vs. poor outcome	P	81	custom array	13.26	$7.46\ 10^{-6}$	Leukemia, Normal karyotype, AML
93	Cheok et al., Nat Genet, 2003, ALL, discrimination of treatments by gene expression	O	107	Affymetrix HG-U95Av2	13.47	$1.05\ 10^{-5}$	Leukemia, ALL
94	Virtaneva et al., Proc Natl Acad Sci U S A, 2001, AML	D	130	Affymetrix HuGeneFl	13.97	$1.29\ 10^{-5}$	Normal karyotype, Chromosomal aberration, Trisomy 8
95	Hofmann et al., Lancet, 2002, Ph+ ALL and prediction of STI571 resistance	O	185	Affymetrix HuGeneFl	13.81	$1.31\ 10^{-5}$	Leukemia, Chromosomal aberration, t9-22, ALL
96	van Delft et al., Br J Haematol, 2005, ALL, AML	D	158	Affymetrix HG-U133A	14.87	$1.51\ 10^{-5}$	ALL, AML
97	Ebert et al., PLoS Med, 2008, MDS, response to lenalidomide	O	159	Affymetrix HG-U133Plus2	17.32	$1.56\ 10^{-5}$	Leukemia, MDS

98	Metzeler et al., Blood, 2008, CN-AML, Survival	P	189	Affymetrix HG-U133 Set	14.36	$3.30\ 10^{-5}$	Leukemia, Normal karyotype, AML
99	van Delft et al., Br J Haematol, 2005, ALL, pre-B, E2A-PBX1	D	69	Affymetrix HG-U133A	11.47	$3.30\ 10^{-5}$	Chromosomal aberration, t(1;19)
100	van Delft et al., Br J Haematol, 2005, AML, FAB M7	D	101	Affymetrix HG-U133A	15.17	$4.59\ 10^{-5}$	
101	Cheok et al., Nat Genet, 2003, ALL, discrimination of treatments by gene expression	O	314	Affymetrix HG-U95Av2	12.69	$4.79\ 10^{-5}$	Leukemia, ALL
102	Valk et al., N Engl J Med, 2004, AML, cluster without predominant characteristics	D	93	Affymetrix HG-U133A	15.85	$4.79\ 10^{-5}$	
103	van Delft et al., Br J Haematol, 2005, ALL, pre-B, hyperdiploid	D	76	Affymetrix HG-U133A	13.08	$5.40\ 10^{-5}$	Hyperdiploid > 50 chromosomes, Hyperdiploid, Chromosomal aberration
104	Verhaak et al., Haematologica, 2009, AML, t(11q23)/MLL	D	54	Affymetrix HG-U133Plus2	12.67	$6.88\ 10^{-5}$	Chromosomal aberration, t(11q23)/*MLL*
105	van Delft et al., Br J Haematol, 2005, ALL, AML	D	156	Affymetrix HG-U133A	11.54	$8.32\ 10^{-5}$	ALL, AML
106	Yeoh et al., Cancer Cell, 2002, ALL, t(1;19)	D	82	Affymetrix HG-U95Av2	12.84	$9.04\ 10^{-5}$	
107	Cheok et al., Nat Genet, 2003, ALL, discrimination of treatments by gene expression	O	106	Affymetrix HG-U95Av2	12.76	$1.13\ 10^{-4}$	Leukemia, ALL
108	Verhaak et al., Haematologica, 2009, AML, KRAS	D	270	Affymetrix HG-U133Plus2	13.24	$1.61\ 10^{-4}$	*KRAS* mutated
109	Cheok et al., Nat Genet, 2003, ALL, discrimination of treatments by gene expression	O	123	Affymetrix HG-U95Av2	11.58	$1.77\ 10^{-4}$	Leukemia, ALL
110	Vey et al., Oncogene, 2004, AML, t(8;21)	D	15	custom array	11.21	$2.70\ 10^{-4}$	Core binding factor aberration, t(8;21), Chromosomal aberration
111	Ross et al., Blood, 2003, B-ALL, hyperdiploid	D	193	Affymetrix HG-U133 Set	9.38	$2.98\ 10^{-4}$	
112	Cheok et al., Nat Genet, 2003, ALL, discrimination of treatments by gene expression	O	213	Affymetrix HG-U95Av2	10.87	$2.98\ 10^{-4}$	Leukemia, ALL
113	Bhojwani et al., J Clin Oncol, 2008, ALL, long-term outcome	P	120	Affymetrix HG-U133Plus2	8.69	$3.09\ 10^{-4}$	Leukemia, B-ALL, pre-B-ALL, ALL
114	Ross et al., Blood, 2004, AML, FAB M7	D	181	Affymetrix HG-U133A	11.43	$4.47\ 10^{-4}$	

115	Valk et al., N Engl J Med, 2004, AML, mostly FAB M4 and M5	D	82	Affymetrix HG-U133A	11.97	5.33 10^{-4}	
116	Yagi et al., Blood, 2003, AML, good vs. poor prognosis	P	90	Affymetrix HG-U95Av2	11.05	5.33 10^{-4}	Leukemia, AML
117	Ferrando et al., Cancer Cell, 2002, T-ALL, TAL1	O	49	Affymetrix HuGeneFl	10.53	5.42 10^{-4}	Leukemia, ALL, T-ALL
118	Ross et al., Blood, 2003, B-ALL vs. T-ALL	D	174	Affymetrix HG-U133 Set	9.21	5.59 10^{-4}	B-ALL, T-ALL
119	Ross et al., Blood, 2004, AML, inv(16)	D	120	Affymetrix HG-U133A	10.79	8.60 10^{-4}	Core binding factor aberration, inv(16), Chromosomal aberration
120	van Delft et al., Br J Haematol, 2005, ALL, T-ALL	D	85	Affymetrix HG-U133A	7.71	1.02 10^{-3}	B-ALL, pre-B-ALL, T-ALL
121	Boultwood et al., Br J Haematol, 2007, MDS 5q- vs. RA normal karyotype	D	144	Affymetrix HG-U133Plus2	7.62	1.02 10^{-3}	Normal karyotype, Chromosomal aberration, del(5q)
122	Valk et al., N Engl J Med, 2004, AML, cluster without predominant characteristics	D	85	Affymetrix HG-U133A	10.60	1.05 10^{-3}	
123	Verhaak et al., Haematologica, 2009, AML, NRAS	D	344	Affymetrix HG-U133Plus2	10.18	1.19 10^{-3}	*NRAS* mutated
124	Cheok et al., Nat Genet, 2003, ALL, discrimination of treatments by gene expression	O	51	Affymetrix HG-U95Av2	9.64	1.28 10^{-3}	Leukemia, ALL
125	Cheok et al., Nat Genet, 2003, ALL, treatment induced gene expression changes	O	59	Affymetrix HG-U95Av2	7.51	1.57 10^{-3}	Leukemia, ALL
126	Cheok et al., Nat Genet, 2003, ALL, treatment induced gene expression changes	O	33	Affymetrix HG-U95Av2	7.98	1.90 10^{-3}	Leukemia, ALL
127	van Delft et al., Br J Haematol, 2005, ALL, pre-B, RUNX1 amplification	D	119	Affymetrix HG-U133A	7.74	1.90 10^{-3}	
128	Boultwood et al., Br J Haematol, 2007, MDS 5q- vs. healthy	D	120	Affymetrix HG-U133Plus2	7.22	2.55 10^{-3}	
129	Cheok et al., Nat Genet, 2003, ALL, discrimination of treatments by gene expression	O	106	Affymetrix HG-U95Av2	6.05	3.41 10^{-3}	Leukemia, ALL
130	Cheok et al., Nat Genet, 2003, ALL, treatment induced gene expression changes	O	48	Affymetrix HG-U95Av2	5.87	5.39 10^{-3}	Leukemia, ALL

131	van Delft et al., Br J Haematol, 2005, ALL, pre-B, hyperdiploid	D	77	Affymetrix HG-U133A	5.94	$6.11\ 10^{-3}$	Hyperdiploid > 50 chromosomes, Hyperdiploid, Chromosomal aberration
132	van Delft et al., Br J Haematol, 2005, ALL, T-ALL	D	61	Affymetrix HG-U133A	6.22	$7.22\ 10^{-3}$	B-ALL, pre-B-ALL, T-ALL
133	van Delft et al., Br J Haematol, 2005, ALL, pre-B, E2A-PBX1	D	142	Affymetrix HG-U133A	5.57	$7.79\ 10^{-3}$	Chromosomal aberration, t(1;19)
134	Ferrando et al., Cancer Cell, 2002, T-ALL, HOX11	O	45	Affymetrix HuGeneFl	5.19	$1.09\ 10^{-2}$	Leukemia, ALL, T-ALL
135	van Delft et al., Br J Haematol, 2005, ALL, pre-B, RUNX1 amplification	D	65	Affymetrix HG-U133A	4.86	$1.32\ 10^{-2}$	
136	Marcucci et al., J Clin Oncol, 2005, AML, ERG	O	39	Affymetrix HG-U133Plus2	5.01	$1.32\ 10^{-2}$	Leukemia, Normal karyotype, AML
137	Yeoh et al., Cancer Cell, 2002, ALL, t(9;22)	D	90	Affymetrix HG-U95Av2	3.74	$1.69\ 10^{-2}$	
138	Vey et al., Oncogene, 2004, AML, t(15;17)	D	13	custom array	−0.27	$5.04\ 10^{-1}$	t(15;17), Chromosomal aberration

Table S5 - *NPM1* mutation in AML with normal karyotype: Ranking of taxonomy terms

Rank	unadjusted p-value	Taxonomy term	Number of signatures	Number of articles
1	< 0.001	*NPM1* mutated	8	4
2	0.028	t(11q23)/*MLL*	9	6
3	0.071	*CEBPA*	7	5
4	0.087	del(7q)	1	1
5	0.113	*FLT3*	6	3
6	0.134	*FLT3*-ITD	2	1
7	0.185	Complex karyotype	1	1
8	0.233	Leukemia	4	3
9	0.233	Healthy	4	3
10	0.332	*CEBPA* double mutant	1	1
11	0.344	3q abnormality	1	1
12	0.350	t(15;17)	4	4
13	0.356	t(8;21)	6	5
14	0.378	Core binding factor aberration	9	5
15	0.475	MDS	1	1
16	0.482	AML	6	4
17	0.490	Chromosomal aberration	37	12
18	0.557	*FLT3*-TKD	2	2
19	0.633	t(6;9)	1	1
20	0.650	inv(16)	2	2
21	0.770	ALL	3	2
22	0.781	del(5q)	3	3
23	0.798	t(11;19)	1	1
24	0.815	Trisomy 8	1	1
25	0.825	t(12;21)	2	1
26	0.829	Normal karyotype	4	4
27	0.880	*KRAS* mutated	1	1
28	0.931	*NRAS* mutated	1	1
29	0.967	t(1;19)	2	1
30	0.968	Hyperdiploid $>$ 50 chromosomes	2	1
31	0.968	Hyperdiploid	2	1
32	0.983	pre-B-ALL	2	1
33	0.993	B-ALL	5	3
34	0.994	T-ALL	4	3

Figure S1 - t(11q23)/*MLL* gene signature from Ross et al.

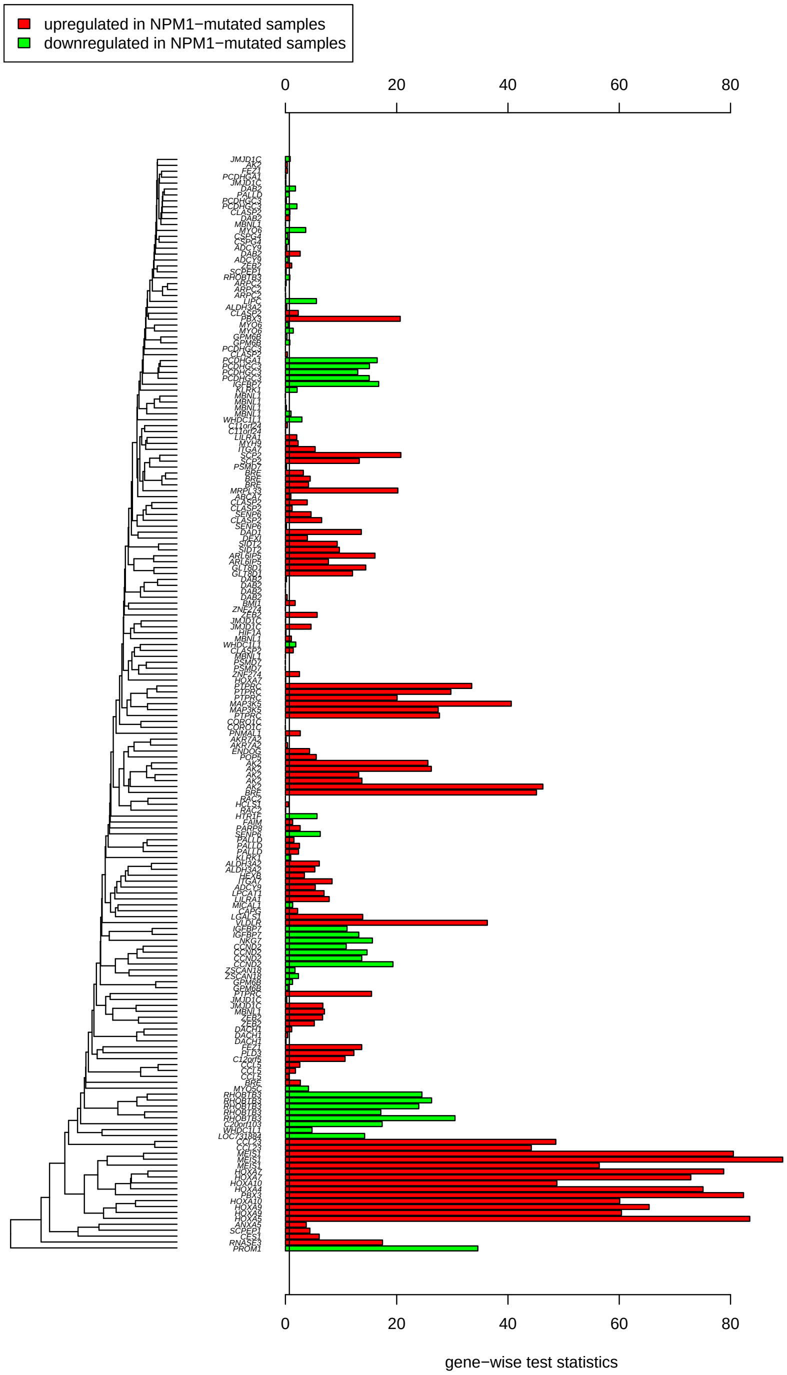

The full plot of all gene wise test statistics that was scaled up for better legibility and partly shown in the manuscript (Figure 3).

Figure S2 - Stability of taxonomy term ranking – Sampling arrays

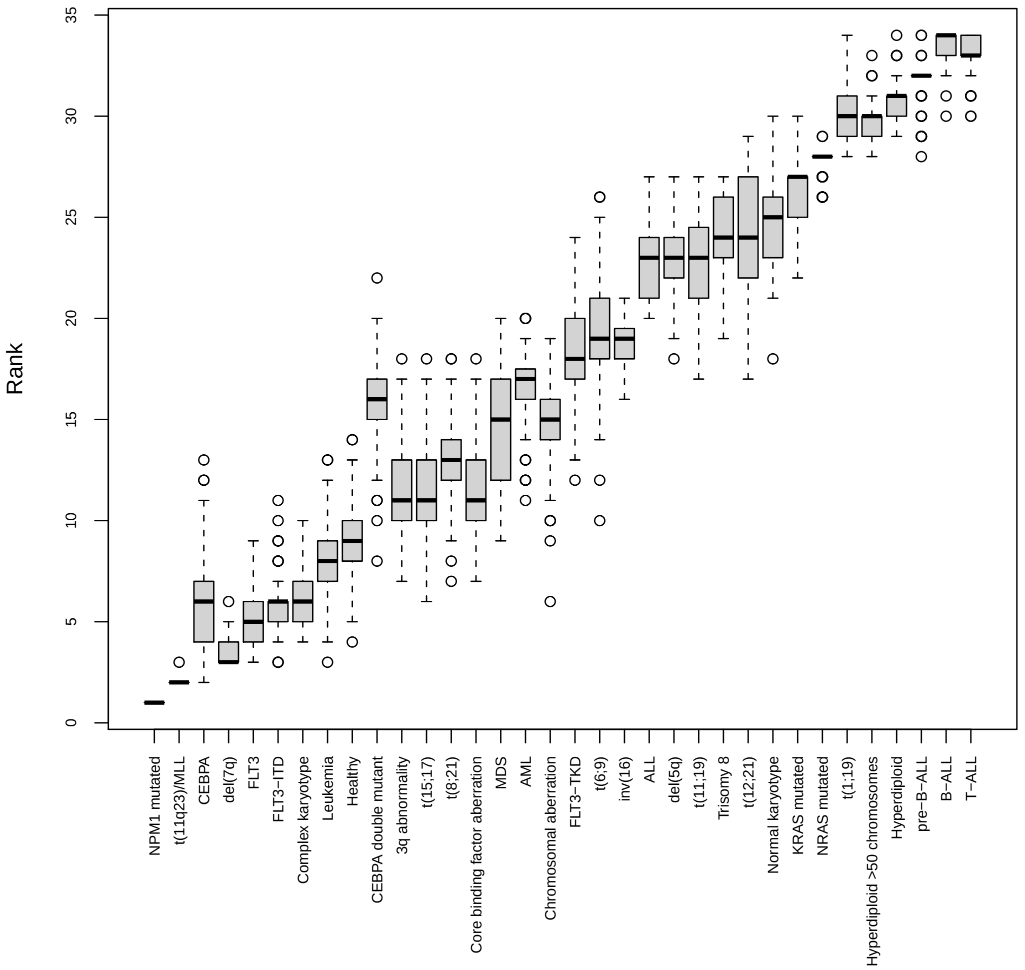

100 subsets of size 126 were drawn out of all 251 arrays to analyze the stability of the results presented in Table S5. The complete analysis including the ranking of taxonomy terms was repeated for each subset. The resulting distributions of the ranks of each taxonomy term are shown in the boxplot. The taxonomy terms are ordered from left to right according to their ranks in the analysis of the full dataset. Due to the lower number of cases, the globaltest's p-values were larger, but the ranking of the gene signatures and thus the ranking of the taxonomy terms remained reasonably stable. Within all 100 runs, the term *NPM1* mutation had unadjusted p-values < 0.001 and was constantly ranked on the first position. Aside from one exception, the translocation t(11q23)/*MLL* was on the second-ranked position with a median p-value of 0.036.

Figure S3 - Stability of taxonomy term ranking – Sampling gene signatures

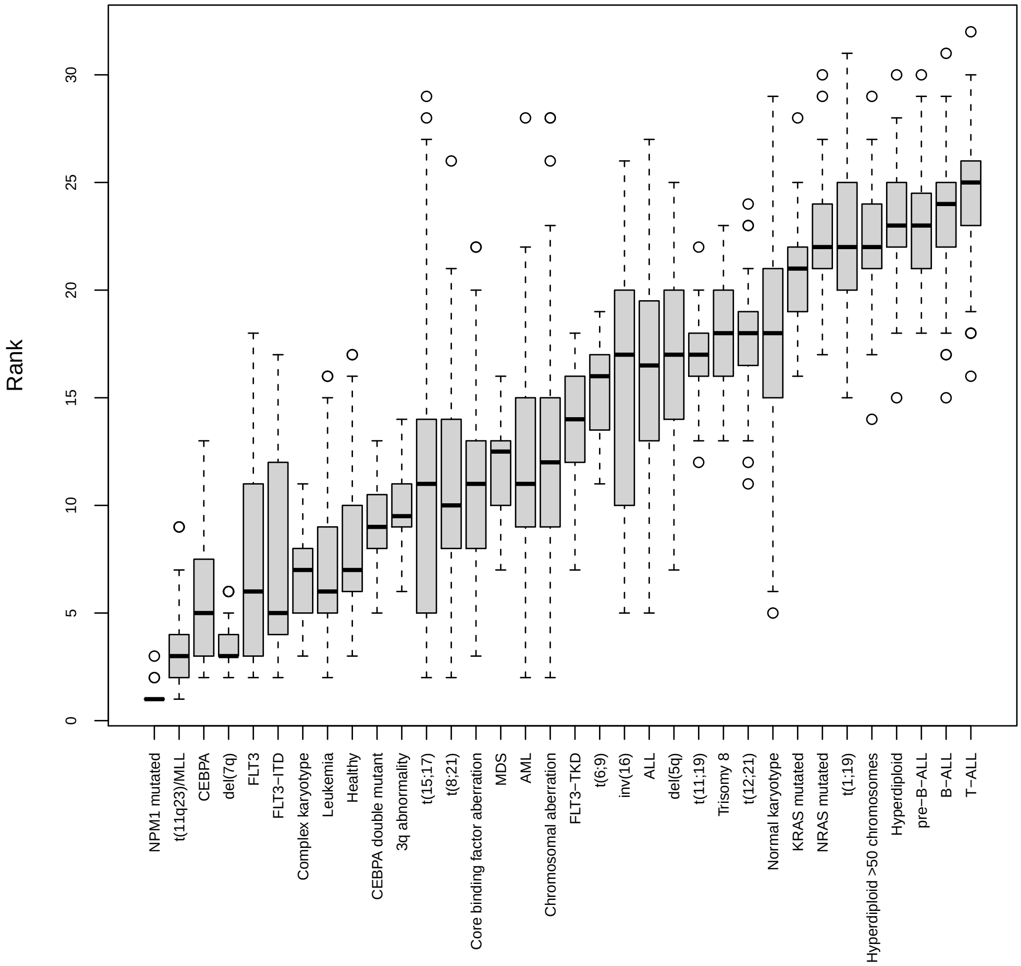

To further assess the stability of the ranking presented in Table S5, 100 subsets of size 69 were taken from all 138 gene signatures in our database. Then, the taxonomy term analysis was repeated for each subset using solely the 69 drawn gene signatures in that set. The boxplot summarizes the observed distribution of the taxonomy terms' ranks. The terms on the x-axis are ordered from left to right according to their ranks based on the complete analysis as presented in Table S5. *NPM1* mutation was nearly always on the first position (three exceptions). The median p-value over 100 runs was 0.001. Translocation t(11q23)/*MLL* ranked 2nd in 44 runs and had a median p-value of 0.075. The increased median p-value (compared to the p-value of 0.028 shown in Table S5) results from the fact that the power to detect an association between t(11q23)/*MLL* and the studied *NPM1* mutation decreases, if half of the gene signatures (likely including some of the 9 signatures associated with t(11q23)/*MLL*) are removed during the sampling process. If a term was not represented by any gene signature in a certain subset, the term was omitted from ranking. This affects the other terms' ranks and thus causes additional variance.
